# Overcoming Biological Barriers: A Comprehensive Review of Advanced Melatonin Delivery Systems for Therapeutic Applications

**DOI:** 10.7150/ijms.134841

**Published:** 2026-06-10

**Authors:** Zhenyu Yang, Yiduo Chen, Shaoying Li, Xiaoyue Lei, Xincheng Wu, Yuan Wang, Shuli Deng

**Affiliations:** 1 Stomatology Hospital, School of Stomatology, Zhejiang University School of Medicine, Zhejiang Provincial Clinical Research Center for Oral Diseases, Key Laboratory of Oral Biomedical Research of Zhejiang Province, Cancer Center of Zhejiang University, Engineering Research Center of Oral Biomaterials and Devices of Zhejiang Province, Hangzhou, 310000, China.; 2 The State Key Laboratory of Fluid Power and Mechatronic Systems & School of Mechanical Engineering, Zhejiang University, Hangzhou 310028, China.

**Keywords:** melatonin, nanocarriers, blood-brain barrier, drug delivery, nose-to-brain delivery, clinical translation

## Abstract

Melatonin is a pleiotropic hormone with well-documented antioxidant, anti-inflammatory, neuroprotective, and immunomodulatory properties, making it a promising candidate for the treatment of diverse diseases including neurodegenerative disorders, cardiovascular diseases, cancer, and sleep disturbances. However, its clinical translation has been hampered by several biopharmaceutical limitations, including poor aqueous solubility, extensive hepatic first-pass metabolism, rapid systemic clearance, and low oral bioavailability. Additionally, physiological barriers such as the blood-brain barrier, stratum corneum, and mucosal epithelia restrict its accumulation at target sites. In recent years, nanotechnology-based drug delivery systems have emerged as powerful tools to overcome these challenges. This review provides a comprehensive overview of advanced melatonin nanocarriers with a focus on their design principles, formulation strategies, and therapeutic applications. A central theme of this review is the integration of carrier design with biological barrier circumvention and administration routes—elucidating how specific nanocarrier platforms address the shortcomings of conventional immediate- and prolonged-release melatonin formulations through spatial and temporal control over drug distribution. We summarize recent preclinical progress in melatonin nanocarriers for a wide range of disease models, including Alzheimer's disease, Parkinson's disease, myocardial infarction, retinal degeneration and glaucoma, depression, and various cancers, with emphasis on the relationship between administration routes and therapeutic outcomes. Finally, critical challenges in clinical translation are addressed, including large-scale manufacturing, long-term toxicity evaluation, regulatory considerations, and the development of chronotherapy-compatible delivery systems. By integrating insights from materials science, pharmaceutics, and nanomedicine, this review aims to provide a rational framework for the future design and clinical application of melatonin-based nanotherapeutics.

## 1. Introduction

### 1.1 Melatonin Biology and Therapeutic Potential

Melatonin (N-acetyl-5-methoxytryptamine) is an evolutionarily conserved indoleamine widely present in bacteria, plants, and vertebrates, where it participates in fundamental biological processes 1, 2. Since its discovery in 1958 3, scientific understanding of this molecule has expanded substantially. Melatonin was initially described as a hormone associated with darkness that regulates the sleep-wake cycle. Subsequent research has demonstrated that it functions as a pleiotropic molecule involved in a broad range of physiological and pathological processes 4. Although the pineal gland represents the principal source of systemic melatonin secretion, substantial synthesis also occurs in peripheral tissues such as the gastrointestinal tract, liver, and skin. In these tissues melatonin may exert local regulatory functions through autocrine or paracrine signaling mechanisms 5.

The diverse biological activities of melatonin are closely related to its distinctive mechanisms of action. As an amphiphilic molecule with both lipophilic and hydrophilic properties, melatonin readily crosses biological membranes and can distribute throughout multiple cellular compartments, including the cytoplasm, nucleus, and mitochondria 6. After entering the cell, melatonin participates in cellular regulation through two major pathways. One pathway involves receptor-mediated signaling. Melatonin binds to membrane receptors melatonin receptor 1 (MT1) and melatonin receptor 2 (MT2) as well as nuclear receptors such as retinoic acid receptor-related orphan receptor alpha (RORα), thereby regulating circadian rhythms, immune responses, and cellular energy metabolism 7. The second pathway involves receptor-independent antioxidant activity. In contrast to conventional antioxidants, melatonin and its metabolites, including N¹-acetyl-N²-formyl-5-methoxykynuramine (AFMK) and N¹-acetyl-5-methoxykynuramine (AMK), participate in a cascade reaction that neutralizes reactive oxygen and nitrogen species (ROS/RNS). Through this cascade mechanism a single melatonin molecule can scavenge multiple free radicals, with some studies suggesting the neutralization of up to ten ROS or RNS molecules 8. In addition, melatonin exerts protective effects directly within mitochondria, contributing to the preservation of mitochondrial function and cellular energy homeostasis 9.

Because of these pleiotropic biological properties, melatonin has attracted considerable attention as a potential therapeutic agent. Experimental and preclinical studies have reported beneficial effects in neuroprotection 10, anti-inflammatory therapy 11, cancer biology 12, metabolic regulation 13, and tissue regeneration 14. However, the translation of these findings into clinical benefit has been inconsistent. Several clinical trials have produced heterogeneous outcomes with significant variability in therapeutic responses. These inconsistencies highlight important limitations associated with the clinical application of melatonin.

### 1.2 Biopharmaceutical Barriers

The limited clinical efficacy of melatonin is not primarily related to the absence of pharmacological activity. Instead, it arises largely from unfavorable physicochemical properties combined with significant pharmacokinetic constraints that restrict the achievement of stable therapeutic concentrations *in vivo* 15.

#### 1.2.1 Physicochemical Properties and Stability Constraints

Melatonin is a relatively lipophilic small molecule with a partition coefficient (log P) of approximately 1.6 and very low aqueous solubility of about 0.1 mg/mL 16. The poor water solubility limits the development of high-concentration formulations and complicates formulation strategies for conventional pharmaceutical dosage forms. In addition, melatonin is sensitive to environmental factors such as light, heat, and oxidative conditions. Exposure to these factors during formulation processing or storage may lead to chemical degradation, which can reduce the stability of pharmaceutical preparations and compromise therapeutic effectiveness 17, 18.

#### 1.2.2 First-Pass Metabolism and Low Bioavailability

Oral administration remains the most common route for melatonin delivery, yet its absolute bioavailability is relatively low and highly variable, typically ranging from 2.5% to 33% 19. A major contributor to this low systemic availability is extensive hepatic first-pass metabolism. After gastrointestinal absorption, approximately 90% of circulating melatonin is rapidly metabolized by hepatic cytochrome P450 (CYP) enzymes, particularly cytochrome P450 1A2 (CYP1A2), to form the inactive metabolite 6-hydroxymelatonin. This metabolite subsequently undergoes conjugation and is eliminated from the body 20. In addition, the acidic environment of the stomach may induce partial degradation of melatonin before absorption occurs, which further decreases the efficiency of oral delivery 21. Another pharmacokinetic limitation is the short plasma half-life of melatonin. Following intravenous administration, the distribution half-life of melatonin is typically 20-40 minutes, reflecting rapid initial clearance 19, 22. Such rapid elimination prevents the maintenance of stable therapeutic concentrations during the night and limits the ability of conventional formulations to reproduce the physiological secretion pattern of endogenous melatonin 22, which exhibits a distinct circadian peak rather than a constant sustained level.

#### 1.2.3 Physiological Barrier Limitations

For therapeutic applications involving central nervous system (CNS) disorders such as Alzheimer's disease (AD) or Parkinson's disease (PD), the blood-brain barrier (BBB) represents a major obstacle to effective drug delivery. Although the lipophilic nature of melatonin theoretically permits passive diffusion across the BBB 23, its accumulation within brain tissue is often insufficient under pathological conditions 24. The BBB is not a uniform barrier; it exhibits regional heterogeneity in transporter expression and tight junction integrity 25. Moreover, disease states such as neuroinflammation can alter BBB permeability in unpredictable ways, sometimes increasing leakiness while simultaneously upregulating efflux transporters 26, 27.

Efflux transporters expressed on BBB endothelial cells, including P-glycoprotein (P-gp), an ATP-dependent efflux pump that recognizes and extrudes a broad spectrum of lipophilic and amphipathic molecules 28. Given its lipophilic nature, melatonin may be susceptible to such efflux mechanisms; the net CNS accumulation of any compound subject to P-gp-mediated transport is determined by the balance between passive influx and active efflux. This efflux mechanism is particularly detrimental when P-gp expression is upregulated, as occurs in drug-resistant epilepsy, certain brain tumors, and chronic neuroinflammation 26. Furthermore, genetic polymorphisms in the ATP-binding cassette subfamily B member 1 (ABCB1) gene encoding P-gp contribute to interindividual variability in transporter activity, which may partially explain the heterogeneous clinical responses to oral melatonin 29. Thus, while passive diffusion across the BBB is theoretically possible, net brain uptake of melatonin is substantially constrained by P-gp-mediated efflux.

A parallel challenge exists for transdermal delivery, which has been investigated as a strategy to bypass hepatic first-pass metabolism. Here, the outermost layer of the skin, the stratum corneum, forms a dense and highly organized barrier that significantly restricts the passive diffusion of most molecules. Consequently, transdermal permeation of melatonin is often limited, and therapeutic levels may not be reliably achieved without formulation strategies designed to overcome this obstacle 30, 31.

**Figure 1:**
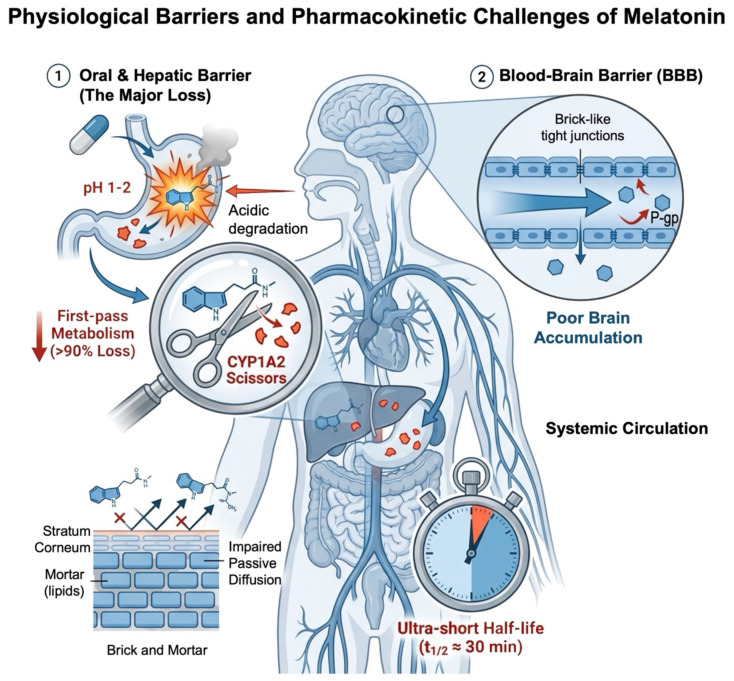
Physiological barriers and pharmacokinetic challenges limiting the clinical efficacy of melatonin. This figure illustrates the key physiological barriers and pharmacokinetic limitations that hinder the clinical application of melatonin. (1) Oral and hepatic barrier represents the major route of drug loss, where melatonin undergoes acidic degradation in the stomach and extensive first-pass metabolism mediated by CYP1A2 in the liver, resulting in over 90% of drug loss. (2) BBB with brick-like tight junctions and P-gp efflux transporters impedes efficient brain accumulation, restricting access to CNS targets. (3) Skin barrier characterized by a stratum corneum brick-and-mortar structure impairs passive diffusion, limiting topical bioavailability. Additionally, melatonin exhibits an ultra-short half-life of approximately 30 minutes, further complicating the maintenance of therapeutic drug levels.

Taken together, these physiological barriers—spanning the BBB, stratum corneum, gastrointestinal tract, and mucosal surfaces—highlight a central theme of this review: the therapeutic efficacy of melatonin is dictated not merely by its intrinsic pharmacological activity, but by the ability of advanced delivery systems to navigate or circumvent these obstacles. The key barriers and pharmacokinetic challenges limiting the clinical application of melatonin are summarized in Figure 1.

### 1.3 Nanotechnology Strategies

To address these challenges, advanced drug delivery systems (DDS) based on nanocarrier technologies have attracted increasing attention as potential strategies for improving the clinical performance of melatonin 4, 32.

Nanotechnology in drug delivery involves more than simple reduction in particle size. Materials engineered at the nanoscale, typically within the range of 1-200 nm, exhibit unique physicochemical properties that can modify the absorption, distribution, metabolism, and elimination of therapeutic agents 33. Encapsulation of melatonin within lipid-based or polymeric matrices can protect the molecule from degradation by digestive enzymes in the gastrointestinal tract and from metabolic enzymes in systemic circulation. Such physical protection improves chemical stability and may prolong the systemic residence time of the drug 4. Moreover, nanoscale particles possess a high surface-area-to-volume ratio. According to the Noyes-Whitney equation, this increased surface area can significantly enhance the dissolution rate of poorly water-soluble compounds. By adjusting the composition and structural characteristics of carrier materials, it is also possible to design delivery systems that exhibit diverse release profiles, ranging from rapid release to sustained release over several days or even weeks.

Functionalized nanocarriers further provide opportunities to overcome biological barriers through targeted delivery strategies. Surface modification with ligands such as transferrin can facilitate receptor-mediated transcytosis across the BBB, thereby enhancing drug transport into the brain. Similarly, the mucoadhesive properties of materials such as chitosan can increase the residence time of drug formulations within the nasal cavity, allowing direct nose-to-brain transport and partially bypassing systemic circulation 34, 35. These active targeting strategies may increase drug accumulation at disease sites while reducing systemic exposure, thereby improving therapeutic efficacy and limiting potential adverse effects 36.

### 1.4 Scope and Objectives of this Review

Existing review articles have often focused either on the pharmacological mechanisms of melatonin or on specific classes of nanocarriers. However, a systematic analysis that integrates the physicochemical properties of carrier materials with the mechanisms of overcoming specific biological barriers remains conspicuously absent. In particular, the relationships among carrier design, structural characteristics, drug loading behavior, and mechanisms for overcoming biological barriers have not been comprehensively analyzed.

The aim of this review is to present a critical assessment of existing melatonin delivery systems, organized according to carrier material classification and examined in the context of their intended administration routes, including oral, intranasal, transdermal, and others. Accordingly, the review first examines the design principles of major carrier platforms, with particular emphasis on how their physicochemical characteristics can be rationally tuned to address distinct barrier challenges, thereby influencing drug loading capacity, formulation stability, and release kinetics. Subsequently, delivery strategies are discussed in relation to specific routes of administration and the physiological barriers they are designed to overcome. Finally, the challenges associated with translating these nanomedicine platforms from laboratory research to clinical application are critically evaluated, including issues related to chemistry, manufacturing, and control, large-scale production, and regulatory considerations. Through this analysis, the review aims to provide a rational framework for the future development and clinical translation of melatonin-based nanotherapeutics.

To ensure a comprehensive and balanced overview of the field, a systematic literature search was conducted using the following electronic databases: PubMed (MEDLINE), Web of Science Core Collection, and Scopus. The search covered publications from January 2000 to December 2025, reflecting the period during which nanotechnology-based delivery strategies for melatonin have been actively developed. Additionally, relevant articles published online ahead of print through March 2026 were included to ensure the review reflects the most current advancements in the field. The primary search strategy employed combinations of the following keywords and Boolean operators: ("melatonin") AND ("nanoparticles" OR "nanocarriers" OR "drug delivery system" OR "liposomes" OR "PLGA" OR "chitosan" OR "lipid nanoparticles" OR "microneedle" OR "intranasal" OR "transdermal"). Additional targeted searches were performed for specific administration routes and disease models (e.g., "nose-to-brain," "ocular delivery," "Alzheimer's disease," "cancer"). This structured approach was intended to minimize selection bias and provide a representative and critical analysis of the current state of melatonin nanocarrier research.

## 2. Lipid-Based Nanocarriers

Lipid-based carriers have become an important platform in nanomedicine because of their strong biomimetic characteristics, favorable biocompatibility, and inherent capacity to encapsulate hydrophobic molecules. These properties make them particularly suitable for the delivery of bioactive compounds such as melatonin 37. The technological progression from classical liposomes to nanostructured lipid carriers (NLCs) reflects a continuous effort to improve drug loading capacity, physical stability, and biological performance. Beyond these improvements, a major objective of this evolution is the ability to regulate drug release kinetics in a spatial and temporal manner so that therapeutic exposure can be aligned more closely with clinical requirements 38.

### 2.1 Liposomes

Liposomes are closed spherical vesicles formed through the self-assembly of phospholipid bilayers, capable of encapsulating both hydrophilic and lipophilic compounds 39. Particle size critically influences their biological fate: smaller vesicles (50-100 nm) evade rapid clearance by the mononuclear phagocyte system, thereby displaying prolonged circulation times 40-42. However, conventional liposomes are relatively rigid and inefficient for transdermal drug delivery 43, and they exhibit limited drug loading capacity and physical instability during storage. These limitations have driven the development of flexible vesicles and next-generation lipid nanocarriers.

### 2.2 Flexible Vesicles

To address the limited transdermal permeability of conventional liposomes, researchers have developed flexible vesicular systems through modification of membrane composition. These systems are designed to enhance membrane fluidity and deformability, thereby improving the ability of vesicles to penetrate the skin barrier.

#### 2.2.1 Transfersomes

Transfersomes are deformable lipid vesicles that incorporate edge activators such as sodium cholate or Polysorbate-80 into the phospholipid bilayer. These surfactants reduce the interfacial tension of the membrane and significantly increase vesicle elasticity 44, 45. As a result, transfersomes are capable of passing through intercellular pores in the stratum corneum that are considerably smaller than the vesicle diameter. Through this deformable transport mechanism, transfersomes can facilitate efficient transdermal delivery of drugs including melatonin, which must overcome the barrier properties of the skin.

#### 2.2.2 Ethosomes

Ethosomes represent another class of flexible vesicles characterized by the presence of high concentrations of ethanol. Ethanol contributes to transdermal permeation through two complementary mechanisms. First, it increases the fluidity of the lipid membrane within the vesicle. Second, it disrupts the ordered lipid arrangement within the stratum corneum, thereby weakening the skin barrier 46. Studies have demonstrated that melatonin-loaded ethosomes significantly increase transdermal flux and reduce the lag time associated with drug permeation. Specifically, melatonin ethosomes with an entrapment efficiency of 70.7 ± 1.4% and a vesicular size of 122 ± 3.5 nm achieved a transdermal flux of 59.2 ± 1.22 μg/cm²/h across human cadaver skin and reduced the permeation lag time to 0.9 h. Confocal laser scanning microscopy confirmed enhanced penetration of the encapsulated payload to a depth of 240 μm within the skin 47. These characteristics make ethosomes an effective strategy for enhancing the transdermal delivery of melatonin and other lipophilic molecules.

### 2.3 Surface Targeting Modifications

In addition to structural optimization of vesicles, surface engineering strategies have been applied to lipid-based carriers to achieve targeted drug delivery. By introducing specific ligands onto the carrier surface, it is possible to direct nanocarriers toward particular tissues or biological barriers. In the context of melatonin delivery, two major targeting strategies have been explored, including brain-targeting systems and skin-targeted delivery.

#### 2.3.1 Brain-Targeting Strategies

The BBB represents a highly selective physiological barrier that restricts the entry of most therapeutic agents into the CNS. To enhance brain delivery, researchers have modified the surface of polyethylene glycol (PEG) -modified liposomes with targeting ligands such as anti-transferrin receptor monoclonal antibodies (e.g., clone OX26) or lactoferrin. These ligands interact with specific receptors expressed on the endothelial cells of the BBB and trigger receptor-mediated transcytosis, allowing the nanocarriers to cross the barrier and deliver drugs into brain tissue, although the efficiency of this process is highly dependent on ligand density, particle size, and surface PEGylation 48-50. This strategy can significantly increase the concentration of melatonin within the CNS and may improve its therapeutic potential for neurological disorders.

#### 2.3.2 Skin-Targeted Delivery

Lipid vesicles interact with the skin through several possible mechanisms. These include direct penetration of intact vesicles through the stratum corneum, disruption or rearrangement of skin lipids that enhances permeation, fusion between vesicle lipids and endogenous skin lipids, and transport through hair follicles 51, 52. In addition, lipid vesicles may create an occlusive effect that increases hydration of the stratum corneum, which in turn facilitates drug diffusion. Due to their favorable biocompatibility and low irritation potential, these systems are particularly suitable for drug delivery to damaged or inflamed skin tissues 53. Consequently, lipid-based vesicles have become an important platform for targeted dermatological therapy. Beyond surface functionalization, modifications to the core structure of lipid carriers have also yielded substantial improvements in pharmaceutical performance.

### 2.4 Solid Lipid Nanoparticles (SLNs)

To overcome the pharmaceutical limitations of traditional liposomes, including drug leakage and residual organic solvents, SLNs were developed as an alternative lipid-based delivery system. SLNs are composed of physiological lipids that remain solid at both room temperature and physiological temperature, forming a solid matrix core capable of encapsulating therapeutic compounds. This solid structure not only provides protection against chemical degradation but also enables SLNs to overcome key biological barriers depending on the administration route. For oral delivery, SLNs can bypass hepatic first-pass metabolism via intestinal lymphatic transport, a pathway whose efficiency depends critically on lipid composition, particle size, and surface hydrophobicity, thereby enhancing systemic bioavailability 54.

In the context of melatonin delivery, SLNs have been shown to significantly improve the photostability of the molecule 55 and provide sustained drug release through oral or transdermal routes. In a clinical pharmacokinetic study involving healthy subjects, oral administration of melatonin-loaded SLN (3 mg) increased the mean area under the curve (AUC) approximately two-fold compared with a standard immediate-release formulation (169,945 ± 64,954 vs. 85,148 ± 50,643 pg/mL·h, *p* = 0.018) and prolonged the elimination half-life from 48.2 ± 8.9 min to 93.1 ± 37.1 min (*p* = 0.009). Transdermal application of the same formulation-maintained plasma levels above 50 pg/mL for at least 24 h, with a mean elimination half-life of 24.6 ± 12.0 h 56. This sustained-release capability, combined with the potential for intestinal lymphatic transport, makes SLNs particularly attractive for oral melatonin delivery when bypassing hepatic first-pass metabolism is a primary objective. Moreover, surface cationization of SLNs has proven effective for ocular delivery, as demonstrated by a single topical application achieving a maximum intraocular pressure reduction of -7 mmHg (p < 0.01) sustained for approximately 24 h in a glaucoma model 57.

However, SLNs are associated with certain technical limitations. During storage, the solid lipid matrix may undergo polymorphic transitions, leading to drug expulsion and an undesirable burst release phenomenon 58. These limitations have stimulated the development of improved lipid carriers with more flexible crystalline structures.

### 2.5 NLCs

NLCs represent an advanced generation of lipid nanocarriers developed to overcome the limitations of SLNs. NLCs are designed based on the concept of imperfect crystal structures. By incorporating liquid lipids into the solid lipid matrix, the highly ordered crystalline structure is disrupted, creating structural imperfections and amorphous regions at the nanoscale 59.

This modified lattice structure provides additional space for drug molecules such as melatonin, thereby increasing the drug loading capacity. At the same time, the presence of structural defects helps prevent drug expulsion during storage and improves the overall physical stability of the formulation 60. Experimental studies have shown that melatonin-loaded NLCs exhibit enhanced skin permeation and improved therapeutic performance compared with free drug formulations 61. As a result, NLCs are considered one of the most promising lipid-based nanocarriers for melatonin delivery. Beyond their improved drug loading and stability, NLCs exhibit excellent performance in nose-to-brain delivery when surface-modified with chitosan, effectively bypassing the blood-brain barrier.

**Figure 2:**
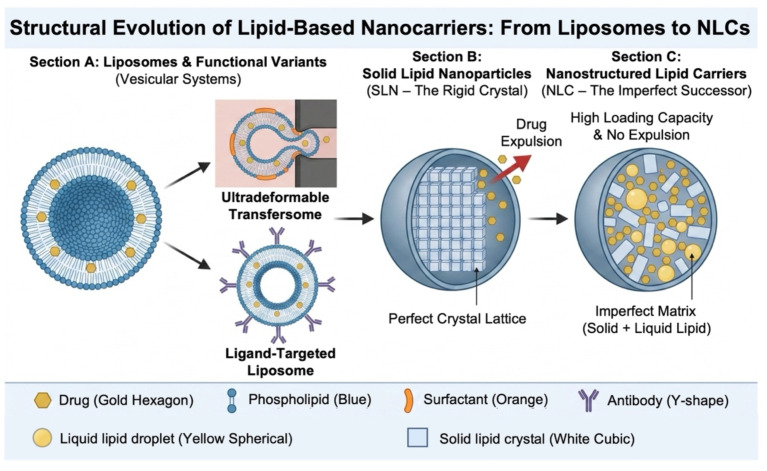
Structural evolution of lipid-based nanocarriers from liposomes to NLCs for melatonin delivery. This figure illustrates the structural evolution of lipid-based nanocarriers, highlighting their design principles and advantages for melatonin encapsulation. Section A presents liposomes and their functional variants. Conventional liposomes are vesicular structures with phospholipid bilayers. Derivatives such as ultradeformable transfersomes enhance skin penetration, while ligand-targeted liposomes enable specific tissue accumulation through antibody conjugation. Section B shows solid lipid nanoparticles, which consist of a perfect crystalline lipid lattice. Although they provide stability, the rigid crystal structure can lead to drug expulsion during storage or phase transition. Section C depicts NLCs, the next-generation system. By incorporating liquid lipids into the solid lipid matrix to form an imperfect lattice, these carriers avoid drug expulsion and achieve higher drug loading capacity, representing an optimized platform for sustained melatonin release.

The structural evolution of lipid-based nanocarriers from liposomes to NLCs carriers for melatonin delivery was summarized and illustrated in Figure 2.

### 2.6 Manufacturing and Excipient Considerations

The performance of lipid-based carriers is not determined solely by their structural design. Manufacturing processes and excipient selection also play critical roles in determining the physicochemical properties and biological performance of the final formulation. Appropriate alignment between preparation methods and carrier structure is therefore essential.

#### 2.6.1 Preparation Method

Because melatonin is sensitive to both light and heat, preparation methods must be selected to minimize degradation during processing. High-pressure homogenization is widely used in industrial production of lipid nanocarriers. For melatonin formulations, the cold high-pressure homogenization method is often preferred. In this approach the drug is first dissolved in molten lipid and rapidly solidified through cooling, followed by mechanical milling at low temperature. This process reduces the exposure of melatonin to prolonged high temperatures that occur in conventional hot homogenization methods and therefore helps preserve drug stability 62, 63.

Supercritical fluid technology has also been investigated as an alternative preparation method. Liposomes prepared using supercritical carbon dioxide have demonstrated encapsulation efficiencies of approximately 82.2% and particle sizes around 66 nm. These formulations exhibit good storage stability and minimal organic solvent residues. *In vitro* digestion studies indicate that such systems can produce an initial sustained release phase followed by complete drug release, which may be beneficial for maintaining therapeutic concentrations 64, 65.

Other preparation methods have also been explored. Microemulsion techniques are relatively simple and do not require high energy input. However, they often require high concentrations of surfactants and co-surfactants to maintain system stability, which may increase the risk of cytotoxicity and limit long-term administration 66. Solvent emulsification-evaporation methods can produce nanocarriers with small particle sizes but require careful control of residual organic solvents. Compared with these approaches, cold high-pressure homogenization and supercritical fluid technology are generally considered more suitable for melatonin lipid formulations.

#### 2.6.2 Key Excipients

The selection of excipients must correspond to the structural characteristics and functional objectives of the carrier system. Lipid matrices represent the first category of critical excipients. Compared with simple fatty acids, complex glycerides can form less ordered crystalline structures, which provide more space for drug incorporation and enhance loading capacity 67. This characteristic is particularly important for NLC formulations designed to create lattice imperfections.

The second category includes surfactants and stabilizers that influence nanocarrier stability and biological distribution. Poloxamer 188, for instance, can reduce uptake by phagocytic cells through steric stabilization, thereby prolonging circulation time in the bloodstream. Polysorbate 80, on the other hand, can adsorb apolipoprotein E on the nanocarrier surface, which facilitates transport across the BBB and supports brain-targeting strategies 68.

A widely adopted strategy for surface modification involves PEG. PEGylation produces so-called stealth liposomes that exhibit improved colloidal stability through steric repulsion, effectively reducing protein adsorption and delaying recognition by the mononuclear phagocyte system. However, the chain length and surface density of PEG must be carefully optimized. While insufficient PEGylation fails to provide adequate protection, excessive PEGylation may trigger immune responses—such as the accelerated blood clearance phenomenon—or alter interactions with biological systems in unintended ways. These issues should be thoroughly evaluated during clinical development 69, 70. Therefore, PEGylation represents a valuable but not universally ideal surface engineering approach.

The third category includes targeting ligands. Molecules such as monoclonal antibodies, peptides, aptamers, or growth factors can be conjugated to the surface of nanocarriers. These ligands enable active targeting by promoting selective accumulation of the drug within diseased tissues 71.

### 2.7 Release Kinetics

The structural evolution and functional modification of lipid-based carriers ultimately manifest in their drug release behavior. For melatonin delivery, the design of an optimal release profile must reconcile two distinct pharmacokinetic imperatives. First, the rapid distribution half-life of the free molecule necessitates a prompt initial release to achieve timely sleep onset 19, 22. Second, maintaining therapeutic plasma concentrations throughout the night requires sustained drug delivery—a goal that prolonged-release formulations address by extending the apparent elimination half-life 72. Importantly, while such formulations offer a prolonged pharmacokinetic profile, this constant-rate delivery should not be conflated with true mimicry of the endogenous circadian rhythm, which is characterized by a dynamic nocturnal peak rather than a steady-state plateau.

The release profile of lipid nanocarriers typically consists of an initial phase in which drug molecules located on or near the particle surface are released rapidly, followed by a slower release phase governed by diffusion and gradual erosion of the lipid matrix 73. This biphasic behavior is often described using the Korsmeyer-Peppas model 74. For spherical nanocarriers, a diffusion exponent value between 0.43 and 0.85 indicates non-Fickian transport, which reflects the combined influence of diffusion and matrix relaxation processes. By adjusting formulation parameters such as the proportion of liquid lipids in NLC systems or introducing surface modifications such as ligand conjugation, it is possible to regulate the diffusion exponent and thereby tailor the release profile to specific therapeutic requirements 75.

## 3. Polymeric Delivery Systems

Lipid-based carriers exhibit strong biomimetic characteristics, whereas polymeric nanocarriers offer advantages in structural stability, controllable degradation kinetics, and extensive opportunities for surface functionalization. These features have established polymeric systems as an important component of modern drug delivery platforms 76. Polymeric systems encompass a versatile range of formulations, including nanocarriers and stimuli-responsive hydrogels. For melatonin delivery, polymer-based carriers can provide sustained drug release and can also be engineered through precise chemical design to form intelligent delivery systems capable of sensing and responding to specific microenvironmental signals 77. Biodegradable polymer nanocarriers generally exhibit particle sizes within the range of approximately 10 to 1000 nm. Drugs can be incorporated into these systems through adsorption, chemical conjugation, or physical encapsulation, thereby protecting the therapeutic compound from degradation while allowing flexible control of pharmacokinetic behavior. The tunable chemical structure of polymeric carriers provides a robust foundation for customized pharmacological applications 78. The key characteristics of major nanocarrier platforms investigated for melatonin delivery, including their formulation materials, key advantages, major limitations, and relevant references, were summarized and illustrated in Table 1.

### 3.1 Poly(lactic-co-glycolic acid) (PLGA) Nanoparticles

PLGA is a synthetic biodegradable polymer that has received approval from the United States Food and Drug Administration (FDA) and has been extensively applied in biomedical research and pharmaceutical formulations 79, 80. Melatonin-loaded PLGA nanoparticles prepared through the emulsion-solvent evaporation method have demonstrated effective sustained-release characteristics 81. A major advantage of this system lies in its ability to modulate degradation behavior and drug release profiles through adjustment of the monomer composition and molecular weight of the polymer.

**Figure 3:**
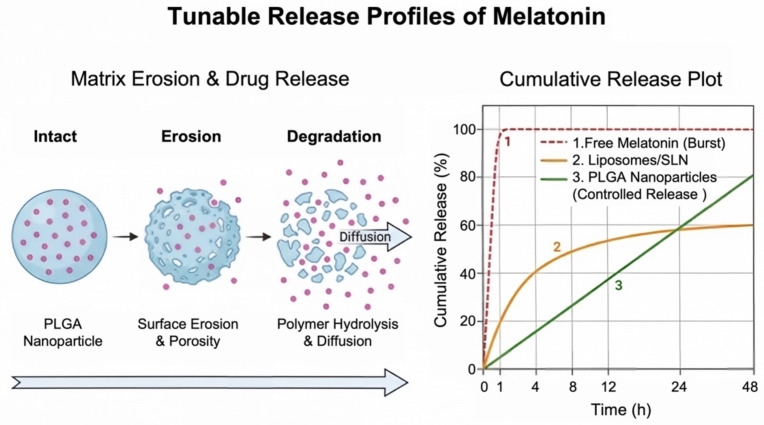
Tunable release profiles of melatonin from diverse delivery systems. This figure demonstrates the tunable drug release kinetics of melatonin enabled by different nanocarrier systems, alongside the underlying release mechanism of PLGA nanoparticles. The left panel illustrates the erosion-mediated release pathway of PLGA nanoparticles, progressing from an intact polymer matrix, through surface erosion and pore formation, to final polymer hydrolysis and drug diffusion. The right panel compares cumulative release profiles: free melatonin exhibits an immediate burst release with nearly complete drug liberation within 1 hour. Liposomes or solid lipid nanoparticles provide intermediate sustained release, achieving approximately 60% cumulative release within 48 hours. PLGA nanoparticles offer linear, controlled release driven by polymer degradation, maintaining sustained drug delivery over 48 hours.

By altering the ratio of lactic acid to glycolic acid, it is possible to control the hydrophilicity and degradation rate of the copolymer 82. Encapsulation of melatonin within PLGA nanoparticles can also reduce degradation caused by gastric acid and digestive enzymes, promote uptake by intestinal microfold cells (M cells), and partially mitigate hepatic first-pass metabolism. These effects collectively contribute to improved systemic bioavailability of the drug 83. The tunable drug release kinetics of melatonin enabled by different nanocarrier systems were summarized and illustrated in Figure 3.

Despite these advantages, PLGA degradation generates acidic by-products such as lactic acid and glycolic acid. Accumulation of these products within the nanocarrier matrix may create a localized acidic microenvironment. This microenvironment can accelerate polymer degradation through autocatalytic processes and may also affect the chemical stability of melatonin 84-86. One strategy to mitigate this issue involves incorporating alkaline buffering agents such as magnesium hydroxide or zinc carbonate into the PLGA matrix. These compounds neutralize the acidic environment generated during polymer degradation and help maintain stable and more predictable drug release kinetics 87.

Beyond degradation control, surface engineering further expands the utility of PLGA nanocarriers. Unmodified PLGA nanoparticles possess hydrophobic surfaces and a negative charge, making them prone to rapid clearance by the mononuclear phagocyte system. PEGylation of PLGA nanoparticles prolongs circulation time and improves systemic stability 88. In addition, reactive functional groups on the PLGA surface enable the conjugation of targeting ligands. For example, folic acid-modified PLGA nanoparticles can selectively target folate receptors overexpressed on many tumor cells 89, promoting preferential accumulation of melatonin in tumor tissues and enhancing antitumor efficacy while reducing systemic adverse effects. Such targeted systems may help exploit the potential of melatonin as a chemotherapeutic sensitizer and immunomodulatory agent.

### 3.2 Chitosan Nanoparticles

Chitosan, a natural polysaccharide derived from the deacetylation of chitin, possesses intrinsic cationic properties, favorable biocompatibility, and biodegradability. These characteristics make it an attractive carrier material for melatonin delivery, particularly in nose-to-brain transport systems 90.

The utility of chitosan in nasal delivery stems from two complementary mechanisms. First, under physiological conditions, the amino groups along the chitosan polymer chain become protonated, generating positive charges that interact electrostatically with negatively charged mucins on the nasal mucosal surface. These positively charged groups can interact electrostatically with negatively charged mucins present on the nasal mucosal surface. This interaction enhances mucoadhesion and prolongs the residence time of the drug formulation at the absorption site, thereby reducing drug loss caused by mucociliary clearance 91. Second, chitosan can also transiently and reversibly open tight junctions between epithelial cells in the nasal mucosa by modulating of proteins such as zonula occludens-1 (ZO-1) 92, 93. It should be noted that the degree of tight junction opening is dependent on chitosan molecular weight, degree of deacetylation, and formulation pH, factors that contribute to variability in delivery efficiency 94. The resulting paracellular transport pathway facilitates the movement of melatonin across the epithelial barrier and into the cerebrospinal fluid, thereby improving the efficiency of nose-to-brain delivery 93.

Experimental studies have validated the potential of chitosan-based melatonin nanocarriers. Chitosan nanoparticles loaded with melatonin have demonstrated good biocompatibility in various cellular models, including human colorectal adenocarcinoma (Caco-2) and Uppsala 87 malignant glioma (U87MG) cell lines, and have shown enhanced cellular uptake, improved antitumor activity, and the ability to promote wound healing 16, 95, 96. Freeze-drying of these nanocarriers with stabilizing agents such as trehalose can further improve their long-term storage stability 97.

### 3.3 Chitosan Derivatives and Composite Systems

Although chitosan possesses favorable biological properties, its solubility is limited to acidic environments. Chemical modification of the polymer has therefore been widely investigated to broaden its applicability and enhance functional performance.

Quaternized chitosan derivatives maintain high water solubility and positive charge across a broad physiological pH range. These derivatives exhibit stronger capacity to open epithelial tight junctions compared with native chitosan, making them suitable for the delivery of hydrophilic drugs and macromolecules 98, 99. Carboxymethyl chitosan, generated through the introduction of carboxyl groups, displays amphoteric characteristics and can undergo self-assembly under specific pH conditions. This property provides opportunities for the development of stimuli-responsive DDS 98, 99.

Chitosan can also be combined with other biomaterials to create multifunctional scaffolds for tissue engineering. For example, composite scaffolds consisting of chitosan and hydroxyapatite have been developed for bone regeneration. When melatonin and bone morphogenetic protein-2 are co-loaded into such scaffolds, the system can simultaneously promote osteogenic differentiation and inhibit osteoclast activity, thereby supporting bone repair processes 100. In another strategy, melatonin and bone morphogenetic protein-2 (BMP-2) can first be encapsulated within PLGA microspheres before incorporation into a chitosan scaffold. This design enables sequential and controlled release of growth factors, which further enhances bone regeneration outcomes 101.

### 3.4 Cyclodextrin-Based Delivery Systems

Cyclodextrins possess a unique molecular structure characterized by a hydrophilic exterior and a hydrophobic internal cavity. This architecture enables cyclodextrins to form host-guest inclusion complexes with hydrophobic molecules such as melatonin, thereby improving the solubility and stability of the drug 102. Cyclodextrin inclusion complexes can be further incorporated into nanocarriers such as PLGA nanoparticles or liposomes to create multi-level delivery systems that combine solubilization with sustained release.

For example, melatonin complexed with hydroxypropyl-β-cyclodextrin can be incorporated into hyaluronic acid hydrogels. In osteoarthritis (OA) models, such systems provide sustained release of melatonin and improve mitochondrial function in chondrocytes 103. In addition, molecular imprinting technology has been explored to further enhance drug delivery performance. By using melatonin as a template molecule during polymerization with cyclodextrin and crosslinking agents such as citric acid, molecularly imprinted nanosponges with selective recognition sites can be produced. Incorporation of these nanosponges into topical cream formulations has been shown to enhance skin permeation and prolong the release of melatonin 104.

### 3.5 Inorganic-Organic Hybrid Systems

Inorganic nanomaterials such as mesoporous silica nanoparticles and metallic nanoparticles possess large specific surface areas, high physicochemical stability, and surfaces that are readily modified. These properties make them suitable components for constructing inorganic-organic hybrid delivery systems.

**Mesoporous silica nanoparticles (MSNs).** MSNs exhibit highly ordered pore structures and substantial drug loading capacity. The pore openings of these particles can be capped with polymers, aptamers, or other gate-forming molecules to create nanoscale gatekeeping systems responsive to environmental stimuli such as pH, redox conditions, or specific enzymes. When exposed to stimuli within the target microenvironment, the gate structures undergo dissociation or structural change, enabling controlled release of the encapsulated drug and reducing premature leakage during circulation 104-107.

**Metallic nanoparticles.** Nanoparticles composed of elements such as gold, palladium, or selenium have also been investigated in combination with melatonin. These hybrid systems have demonstrated biological effects that exceed those of the individual components in several experimental models. For instance, gold nanoparticles combined with melatonin have shown enhanced anti-inflammatory and antioxidant effects in models of testicular injury 108. Palladium nanoparticles used in combination with melatonin have been reported to promote apoptosis in lung cancer cells 109. Gold nanoparticles also possess photothermal conversion properties, enabling the integration of photothermal therapy with controlled drug release strategies 110.

**Stimuli-responsive hydrogels.** As an important class of intelligent hybrid materials, stimuli-responsive hydrogels can undergo swelling, degradation, or phase transitions in response to environmental signals such as pH, ROS, or specific enzymes. Such responses enable precise control of drug release behavior. Hydrogels designed to respond simultaneously to acidic pH and elevated oxidative stress characteristic of tumor microenvironments can achieve site-specific release of melatonin through programmed degradation processes 111, 112.

Collectively, the integration of inorganic-organic hybrid materials with stimuli-responsive strategies represents a promising direction for the development of precise and personalized melatonin delivery systems. Such platforms hold the potential to address multiple physiological barriers simultaneously—for example, facilitating blood-brain barrier penetration while enabling triggered intracellular drug release at the target site.

## 4. Administration Routes for Melatonin

The pleiotropic biological activities of melatonin require diverse administration routes to accommodate a wide range of clinical applications, including sleep regulation, neuroprotection, anti-inflammatory therapy, and tissue protection. Conventional oral administration is significantly affected by extensive first-pass metabolism, which restricts systemic bioavailability. Consequently, the design of delivery strategies capable of overcoming specific anatomical and physiological barriers has become an important component in bridging fundamental research with clinical translation.

### 4.1 Nose-to-Brain Delivery

Nose-to-brain delivery has emerged as a promising strategy for transporting therapeutic agents directly to the CNS. This approach is designed to bypass physiological barriers such as the BBB and facilitate direct drug transport into brain tissue. Several pharmaceutical products delivered through the nasal route, including vaccines, analgesics, antimigraine drugs, anticancer agents, and hormone therapies, have entered clinical development or commercialization stages 113. As a noninvasive brain-targeting method, intranasal administration has attracted considerable interest for the treatment of neurological disorders including PD and AD 114.

#### 4.1.1 Anatomical Pathways

Following intranasal administration, drugs may reach the brain through two principal anatomical pathways. The first is the olfactory pathway. In this mechanism, drug molecules are absorbed by the olfactory epithelium and transported along the axons of olfactory neurons toward the olfactory bulb. From there, the molecules may enter the cerebrospinal fluid and subsequently distribute to various brain regions 115, 116. The second pathway involves the trigeminal nerve system. Branches of the trigeminal nerve innervating the nasal cavity are capable of internalizing drug molecules and transporting them toward the brainstem and pons 114. Through these pathways, drugs can bypass systemic circulation and avoid the restrictive properties of the BBB, thereby reaching CNS targets more efficiently, though the extent of nose-to-brain transport is highly formulation-dependent, influenced by factors such as particle size, surface charge, and mucoadhesive properties 117, 118.

Despite these advantages, nose-to-brain delivery is associated with several limitations. The nasal cavity has a relatively small volume, and the mucociliary clearance mechanism rapidly removes foreign substances. Additional challenges include enzymatic degradation within the nasal mucosa, short drug residence time, potential mucosal irritation, and the need for specialized delivery devices capable of ensuring accurate deposition in the upper nasal region 118, 119.

#### 4.1.2 Nanocarrier Applications

**Figure 4:**
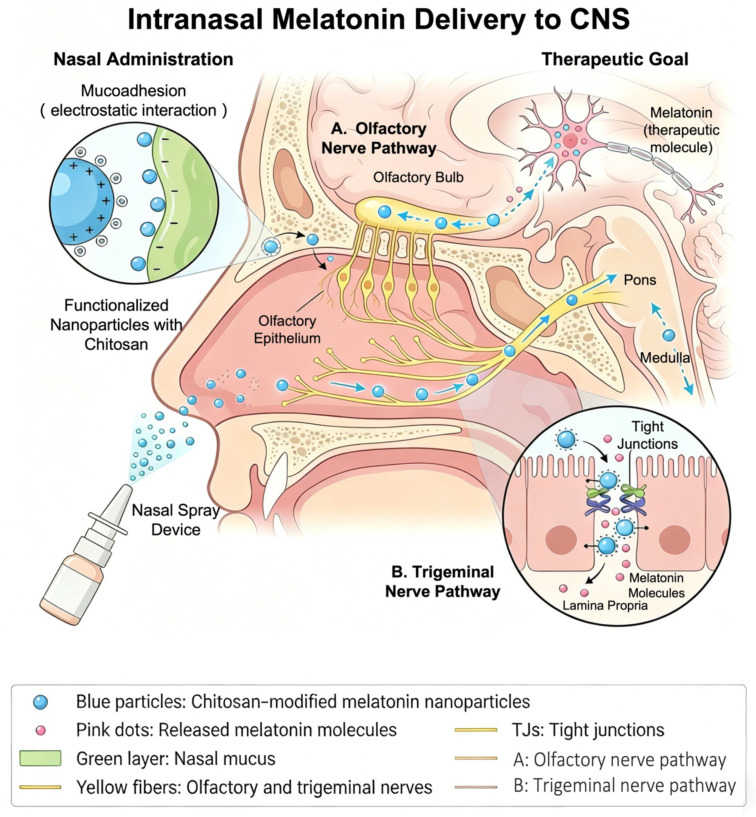
Intranasal melatonin delivery to the CNS via chitosan-modified nanocarriers. This figure illustrates the mechanism of intranasal melatonin delivery to the CNS using chitosan-functionalized nanoparticles. Chitosan-coated nanoparticles are administered via a nasal spray device. The positively charged chitosan enables mucoadhesion through electrostatic interactions with the negatively charged nasal mucus, prolonging the residence time of the formulation. The nanoparticles traverse the nasal epithelium via two main pathways: the olfactory nerve pathway, which transports drug molecules to the olfactory bulb and further into the brain, and the trigeminal nerve pathway, which delivers drug to the pons and medulla. Additionally, the nanoparticles can reversibly open epithelial tight junctions, creating paracellular shunts that enhance drug penetration into the CNS, thereby bypassing the systemic circulation and BBB for targeted central therapeutic effects.

To exploit the anatomical advantages of intranasal administration, delivery systems must possess adequate mucosal adhesion and permeation capability. Nanotechnology has therefore become a key formulation strategy in this field 119-121. Cationic chitosan coatings can enhance the electrostatic interaction between nanocarriers and negatively charged nasal mucosa, thereby increasing mucosal adhesion. Chitosan can also reversibly open tight junctions between epithelial cells, which improves paracellular transport and increases drug concentrations in the brain. Furthermore, the positively charged nature of chitosan allows interaction with negatively charged epithelial surfaces or promotes hydration of the mucus layer, forming a gel-like barrier that prolongs residence time within the nasal cavity 122, 123. The mechanism of intranasal melatonin delivery to the CNS using chitosan-functionalized nanocarriers was summarized and illustrated in Figure 4.

Melatonin-loaded polycaprolactone nanocarriers administered intranasally have demonstrated significantly higher cytotoxic activity against glioblastoma cells compared with free melatonin, with an inhibitory concentration (IC₅₀) value approximately 2500-fold lower than that of free melatonin indicating improved brain-targeting efficiency 124. Similarly, melatonin-loaded lipidic nanocapsules (LNCs) exhibited 10.35-fold higher permeation across sheep nasal mucosa compared with drug solution, and post-ischemic intranasal administration significantly reduced oxidative stress markers while restoring hippocampal neurons in a cerebral ischemia/reperfusion model 125. In addition, melatonin-dopamine derived nanocomposites exhibiting near-infrared responsiveness have been reported to inhibit β-amyloid aggregation and alleviate oxidative stress and inflammatory responses in models of AD 126.

Preclinical studies have further demonstrated that intranasal administration of melatonin nanocarriers can produce pronounced sleep-inducing effects, with higher brain targeting efficiency than intravenous administration 127. However, this delivery route still faces several challenges, including dose limitations, local tolerability concerns, and potential pulmonary exposure associated with aerosolized formulations 128.

### 4.2 Pulmonary Inhalation Delivery

The lung represents an attractive target for systemic drug delivery because of its large absorption surface area and extremely thin air-blood barrier. These characteristics enable rapid drug absorption into the systemic circulation and are particularly relevant for the treatment of pulmonary disorders such as acute lung injury 129. Aerodynamic properties are critical determinants of drug deposition within the respiratory tract. For efficient deep lung deposition and systemic absorption, inhalable particles generally require a mass median aerodynamic diameter between 1 and 5 μm 130.

Large porous particle technology has been developed to optimize pulmonary deposition. These particles possess relatively large geometric diameters but extremely low density, allowing them to deposit efficiently in the deep lung while avoiding rapid phagocytosis by alveolar macrophages 131. Pharmacokinetic modeling suggests that inhalation of 2 mg melatonin could theoretically produce plasma concentrations up to 26.8 times higher than those achieved through oral administration at the same dose, highlighting the high efficiency of pulmonary delivery 132.

Melatonin large porous particles produced by spray-drying technology have demonstrated rapid systemic absorption following inhalation while maintaining elevated local drug concentrations in the alveolar region. Through direct neutralization of ROS, these formulations may effectively reduce inflammation and oxidative stress associated with acute lung injury 133.

### 4.3 Oral Delivery Systems

Despite the challenges associated with oral administration, this route remains the most widely used in clinical practice because of its convenience and high patient compliance. Oral melatonin formulations are particularly relevant for sleep management in patients with neurodegenerative diseases 134. However, the bioavailability of orally administered melatonin remains relatively low, typically around 15%, with considerable interindividual variability. These limitations arise from inconsistent gastrointestinal absorption and extensive hepatic first-pass metabolism 19.

The application of nanotechnology to oral melatonin formulations has primarily focused on improving drug solubility and metabolic stability. Gastric retention systems represent one strategy for enhancing oral delivery. Floating systems or bioadhesive microspheres can prolong the residence time of the drug in the stomach, enabling sustained drug release. Such systems may be beneficial for exploiting the gastroprotective effects of melatonin in conditions such as gastric ulcers 135.

Colon-targeted delivery systems represent another strategy. These formulations typically employ pH-sensitive polymer coatings that remain stable in the acidic environment of the stomach but dissolve in the alkaline environment of the colon. As a result, the drug is released specifically in the colon, thereby avoiding enzymatic degradation in the upper gastrointestinal tract. This approach has potential therapeutic relevance for diseases such as ulcerative colitis 136.

Although several sustained-release melatonin tablets have already been introduced into the market, oral therapy continues to be limited by the intrinsic constraints of the gastrointestinal route. It is noteworthy that certain inhalable melatonin products lacking comprehensive safety and efficacy evaluation have appeared in commercial markets, and their clinical value requires further validation.

### 4.4 Buccal and Sublingual Delivery

Drug delivery through the oral mucosa allows direct absorption into the internal jugular vein, thereby bypassing hepatic first-pass metabolism. This route is particularly advantageous in clinical situations requiring rapid onset of action. For conditions such as sleep onset insomnia, the speed at which therapeutic effects are achieved is critical. Sublingual administration offers several advantages including avoidance of first-pass metabolism, ease of administration, suitability for elderly patients or individuals with swallowing difficulties, and minimal mucosal irritation 137.

Melatonin-loaded sublingual nanofiber formulations have demonstrated extremely rapid disintegration properties. Some systems are capable of disintegrating within 1 second and dissolving completely in phosphate buffered saline that simulates saliva within 90 seconds 138. The choice of carrier material is crucial for maintaining the structural integrity, functionality, and overall performance of nanofibers 139. Polyvinylpyrrolidone K90 has been widely investigated because of its hydrophilicity, low toxicity, biocompatibility, and bioadhesive properties. These characteristics enable prolonged residence of the formulation on the mucosal surface and make it a suitable carrier for sublingual nanofiber systems 140.

Research has also indicated that sublingual nanofiber formulations containing melatonin may enhance the therapeutic efficacy of chemotherapeutic agents such as doxorubicin, suggesting potential applications in combination therapy for cancer treatment 141.

To achieve prolonged drug release through oral mucosal administration, innovative formulation strategies have been proposed. One approach involves integrating sustained-release drug microparticles into rapidly dissolving oral films. For example, drug-polymer microparticles prepared through hot-melt extrusion can be incorporated into solvent-cast orally dispersible films. This design significantly prolongs drug release and may be particularly useful for maintaining sleep throughout the night 142, 143.

### 4.5 Microneedle-Based Transdermal Systems

The skin represents an attractive interface for long-acting drug delivery; however, the stratum corneum forms a formidable physiological barrier that restricts passive drug permeation. Microneedle technology offers a means to overcome this obstacle 144, 145. By creating transient microchannels across the stratum corneum, microneedle-based systems enable melatonin to access the dermal microcirculation, achieving systemic delivery while inherently bypassing hepatic first-pass metabolism—a primary cause of melatonin's low and variable oral bioavailability. Furthermore, these systems can sustain near-constant plasma levels for 4-8 hours 146, 147, maintaining therapeutic concentrations throughout a typical sleep period. This extended coverage meets a clinical need that oral immediate-release formulations cannot fulfill and that current oral prolonged-release products only partially address, given their apparent elimination half-life of approximately 4-5 hours 72. Such advantages hold particular relevance for elderly patients with swallowing difficulties, individuals with circadian rhythm sleep disorders who require reliable overnight coverage without repeated dosing, and those seeking a non-injectable alternative for systemic melatonin therapy.

Depending on therapeutic objectives, transdermal delivery may serve either local dermatological effects or systemic absorption; for melatonin, systemic absorption constitutes the primary research focus 144. Microneedle arrays consist of hundreds of microscopic projections that painlessly penetrate the epidermis, forming transient channels that permit drug diffusion into the dermal capillary network. This approach combines the high bioavailability of subcutaneous injection with the convenience and patient acceptance of a transdermal patch 145.

#### 4.5.1 Silk Fibroin Microneedles

Among various microneedle matrix materials, silk fibroin has attracted considerable interest because of its excellent biocompatibility, favorable mechanical strength, and controllable degradation behavior. These characteristics make it a promising carrier for melatonin delivery 146.

Silk fibroin microneedle patches loaded with melatonin have demonstrated promising therapeutic outcomes in experimental insomnia models. In one study, silk fibroin microneedles were capable of releasing melatonin continuously for more than 11 hours with a cumulative release exceeding 85%. In rat models of insomnia, therapeutic plasma concentrations were maintained for approximately eight hours, resulting in improved sleep architecture and reduced anxiety-like behavior 147. Complementary work confirmed rapid systemic absorption—peak plasma concentration above 10 ng/mL at 0.31 h—and sustained levels exceeding 5 ng/mL for four to six hours 148.

This sustained-release profile effectively prolongs systemic exposure and maintains therapeutic drug levels over an extended period, which represents a pharmacokinetic advantage over immediate-release formulations. However, it is important to note that this constant-rate delivery does not truly recapitulate the dynamic, pulsatile circadian rhythm of endogenous pineal melatonin secretion, which is characterized by a distinct nocturnal peak followed by a gradual decline toward morning. Therefore, while silk fibroin microneedles offer a clinically useful prolonged-release profile for overnight sleep maintenance, they constitute a pharmacokinetic prolongation strategy rather than a true chronotherapeutic system that dynamically adapts to circadian phase.

#### 4.5.2 Microneedle Types and Manufacturing

Beyond dissolvable silk fibroin microneedles, other microneedle configurations have been developed to address different clinical needs 149. Hollow microneedles possess a structure similar to miniature syringes and can rapidly inject drug solutions, enabling quick elevation of systemic drug levels. This approach may be suitable for acute interventions requiring rapid sleep induction 150. Coated or drug-loaded microneedles concentrate the drug at the needle tip and release it rapidly after skin insertion, which is beneficial for treatments requiring rapid pharmacological action 151, 152.

Advances in three-dimensional printing technology have further expanded the design possibilities of microneedle systems. Additive manufacturing techniques allow precise control over microneedle height, geometric shape, array density, and internal porosity. These parameters can be adjusted to regulate drug release kinetics, providing opportunities for the development of personalized dosing strategies 153-155.

### 4.6 Ocular Nanodelivery Strategies

Melatonin has demonstrated potential therapeutic effects in ocular disorders, including intraocular pressure (IOP) reduction and retinal protection. Unlike transdermal delivery, where systemic absorption is often the primary goal, ocular melatonin delivery is almost exclusively directed toward local tissue retention—prolonging residence time on the corneal surface or within the precorneal region to achieve sustained intraocular effects while minimizing systemic exposure 57.

However, conventional eye drops exhibit extremely low bioavailability because of rapid tear clearance and the barrier properties of the corneal epithelium. To overcome these limitations, nanotechnology-based ocular delivery systems have been developed along two principal directions. One approach involves surface engineering to enhance adhesion and penetration at the ocular surface. The other strategy employs in situ gelling systems that form sustained drug reservoirs following administration.

#### 4.6.1 Surface-Engineered Nanocarriers

Surface modification of nanocarriers can significantly improve interactions with the negatively charged corneal surface, thereby prolonging ocular residence time. For example, cationic SLNs modified with didodecyldimethylammonium bromide exhibit strong electrostatic adhesion to the corneal surface. A single topical application of this formulation achieved a maximum intraocular pressure reduction of -7 mmHg (p < 0.01) in albino rabbits, with the hypotensive effect sustained for approximately 24 h 57.

PLGA-PEG nanoparticles have also demonstrated sustained IOP reduction for approximately eight hours in rabbit eyes as a result of optimized particle size and hydrophilic surface modification 156. Similarly, hybrid lipid-polymer nanocarriers combining the biomimetic properties of liposomes with the structural stability of polymeric cores have been developed to enhance ocular mucoadhesion and prolong pre-corneal residence time, thereby improving the ocular bioavailability of melatonin 157.

#### 4.6.2 In Situ Gel Systems

In addition to surface modification strategies, in situ gel systems represent another important approach for ocular drug delivery. These formulations are administered as liquid eye drops but undergo rapid phase transition into semi-solid gels when exposed to physiological conditions such as body temperature or ionic composition of tear fluid. The resulting gel structure resists tear drainage and forms a localized drug reservoir in the precorneal region, enabling sustained drug release.

Studies incorporating melatonin-loaded nanocapsules into thermosensitive or ion-sensitive gels have demonstrated delayed drug release and improved corneal permeation, as systematically reviewed by Romeo et al. in the context of ocular melatonin delivery 156, 158. These systems provide a promising platform for prolonged ocular therapy and improved treatment outcomes.

### 4.7 Comparative Analysis: Linking Carrier Design to Biological Barriers

The therapeutic efficacy of melatonin is ultimately determined not by the isolated merits of a carrier or a route, but by the precise alignment between carrier properties, barrier challenges, and administration strategies. To crystallize this principle, the following analysis directly links the physicochemical characteristics of each major carrier platform to the biological barriers they are best equipped to overcome and the routes through which they are most effectively deployed.

#### 4.7.1 Bypassing Hepatic First-Pass Metabolism

To minimize extensive CYP1A2-mediated degradation in the liver, carriers must either avoid portal vein absorption or protect the drug during hepatic transit. Among the systems reviewed, lipid-based carriers (SLNs and NLCs) offer a distinct advantage for oral delivery via intestinal lymphatic transport, effectively shunting melatonin around the liver and into systemic circulation via the thoracic duct 159. Additionally, transdermal microneedle systems and buccal/sublingual nanofibers bypass the gastrointestinal tract and hepatic portal system entirely, providing the most direct circumvention of first-pass metabolism 160. In contrast, conventional PLGA nanoparticles, while offering sustained release, are absorbed primarily into portal blood and thus afford only partial protection against hepatic clearance unless surface-engineered for lymphatic targeting 83.

#### 4.7.2 Enabling Brain Delivery

For CNS targeting, the ability to cross or bypass the BBB is paramount. Chitosan-based nanoparticles are uniquely suited for nose-to-brain delivery owing to their mucoadhesive properties and capacity to reversibly open tight junctions in the nasal epithelium, enabling direct olfactory and trigeminal nerve transport 123. Among lipid-based systems, PEGylated liposomes functionalized with brain-targeting ligands such as transferrin or rabies virus glycoprotein (RVG) peptide represent the most plausible intravenous strategy for receptor-mediated transcytosis across the BBB 161. While PLGA nanoparticles can sustain drug release, their intrinsic brain penetration is limited unless actively targeted; their primary utility in CNS applications lies in local intracranial implants or intranasal delivery rather than systemic brain targeting 83, 162.

#### 4.7.3 Clinical Translation Potential

From a regulatory and manufacturing standpoint, PLGA nanoparticles and SLNs currently hold the greatest translational promise. Both systems benefit from Generally Recognized as Safe (GRAS) excipients, established large-scale manufacturing protocols, and extensive preclinical toxicology data 163. Chitosan nanoparticles face additional hurdles related to batch-to-batch variability in molecular weight and degree of deacetylation, though their bioadhesive advantages for mucosal delivery remain compelling 94. Hybrid inorganic-organic systems and chronobiology-adapted smart hydrogels represent exciting but early-stage technologies; their translational timeline is considerably longer owing to unresolved questions regarding long-term accumulation, immunogenicity, and complex manufacturing workflows 111, 164.

This integrative logic extends beyond these representative barriers to the stratum corneum, ocular surface, and pulmonary mucosa, as systematically summarized in Table 2.

## 5. Clinical Translation and Future Directions

Melatonin has transcended its traditional role as a sleep-regulating hormone and demonstrates broad therapeutic potential as an endogenous antioxidant, immune modulator and neuroprotective agent.

Current clinical melatonin formulations fall into two principal categories: immediate-release (IR) and prolonged-release (PR) preparations. IR products (e.g., Adaflex®, Ceyesto®, Syncrodin®, and Bio-Melatonin) are rapidly absorbed, producing a transient plasma peak that facilitates sleep onset, but their short elimination half-life (20-40 min) precludes sustained overnight coverage. PR formulations, typified by Circadin® (2 mg prolonged-release tablets, EMA-approved for primary insomnia in adults aged ≥55 years), extend melatonin release over 8-10 hours via a matrix-type delivery system, thereby prolonging the apparent elimination half-life to approximately 4-5 hours 165. Slenyto® 166 represents a pediatric PR formulation specifically developed for sleep disorders in children with neurodevelopmental conditions. Despite these advances, both IR and PR formulations share fundamental limitations inherent to oral administration: extensive and variable first-pass metabolism (resulting in absolute bioavailability of only 2.5%-33%), inability to target specific anatomical compartments beyond the systemic circulation, and an invariant release profile that cannot recapitulate the dynamic circadian peak characteristic of endogenous pineal secretion.

In contrast, the nanocarrier platforms reviewed herein offer potential solutions to these shortcomings through capabilities that conventional oral formulations are not equipped to address. These include bypassing hepatic first-pass metabolism via lymphatic transport, achieving sustained release over weeks to months, circumventing the blood-brain barrier for direct CNS delivery, and enabling transdermal or ocular administration with prolonged local or systemic retention. Collectively, these platforms expand the therapeutic reach of melatonin from mere systemic exposure prolongation to true spatial and temporal control over drug distribution.

Representative preclinical studies investigating these nanocarrier systems across diverse disease models are summarized in Table 3. Studies included in Table 3 were selected based on the following criteria: (i) studies reporting the application of a specifically engineered nanocarrier for melatonin delivery; (ii) preclinical *in vivo* studies or advanced *in vitro* disease models with quantitative therapeutic outcome measures; and (iii) studies published in English in peer-reviewed journals. Studies focusing solely on the pharmacological mechanism of melatonin without a delivery system component, or studies using only conventional immediate-release formulations, were excluded. Despite the encouraging preclinical data summarized in Table 3, the clinical translation of melatonin nanocarriers faces several interconnected challenges.

### 5.1 Scalable Manufacturing and Batch Consistency

Current melatonin dosage forms include tablets, capsules and topical formulations 81, while nanocarrier-based melatonin delivery systems remain at an early exploratory stage 177. Most high-performance nanocarriers, such as functionalized PLGA nanoparticles and drug-loaded microneedles, are still produced at milligram scale in laboratories. During industrial scale-up, issues such as broadened particle size distribution and reduced encapsulation efficiency commonly arise, impairing product quality and stability.

Microfluidic technology offers critical support for a smooth transition from laboratory studies to Good Manufacturing Practice (GMP)-compliant pharmaceutical manufacture. By precisely controlling fluid mixing and reaction processes within micron-scale channels, microfluidics can continuously and reproducibly produce nanomedicines with narrow and uniform particle size distributions, facilitating batch-to-batch consistency required by stringent pharmaceutical quality systems 64. In continuous production studies of melatonin self-nanoemulsifying drug delivery systems (SNEDDS), nanocarriers prepared using 3D-printed microfluidic chips exhibited particle size distributions and key quality attributes comparable to those from conventional batch methods, indicating that microfluidic technology can maintain strict quality standards while improving production efficiency 177. Future work must progress from proof-of-concept to establishment of robust, scalable production platforms, focusing on integrated continuous processes that combine nanocarrier formation, purification and final formulation steps, implementing process analytical technologies for real-time monitoring of critical quality attributes, and defining design-space parameters that specify acceptable ranges for process variables. These efforts are essential to translate laboratory innovations into commercially viable products.

### 5.2 Long-Term Toxicology and Biocompatibility

Melatonin itself has a favorable safety profile; exogenous supplementation causes mild adverse effects and does not induce dependence 178, 179. However, the long-term safety of nanocarrier materials remains a central concern for clinical translation. Following intravenous administration, nanocarriers are readily sequestered by the mononuclear phagocyte system and distributed to organs such as the liver and spleen; surface hydrophilic modifications can reduce clearance but may alter biodistribution characteristics.

Encapsulating melatonin in biocompatible carriers can prevent drug oxidation, reduce potential toxicity and improve pharmacokinetic profiles 180. Nevertheless, many novel nanomaterials, such as metal-organic frameworks and complex surfactant systems, have unclear long-term accumulation, immunogenicity and metabolic fates *in vivo*. For indications requiring chronic administration, including neurodegenerative diseases, the risk of chronic accumulation in the brain requires careful assessment. A shift is therefore needed from efficacy-centered evaluation toward integrated efficacy-toxicology assessment. Advanced *in vivo* tracing techniques, including radiolabeling and multimodal imaging, should be employed to systematically investigate nanocarrier biodistribution, degradation pathways and elimination kinetics across their biological lifecycle. Concurrently, predictive *in vitro* models simulating chronic exposure scenarios should be developed, and structure-activity relationships linking nanocarrier physicochemical properties to toxicological outcomes should be established. Exploration of biomimetic carriers derived from endogenous components (e.g., exosomes, albumin, cell membrane-coated nanoparticles) may provide inherently safer alternatives 75. These studies will generate the rigorous safety data required for regulatory approval.

### 5.3 Smart Responsive and Chronobiology-Adapted Systems

Melatonin exerts physiological effects that are strongly dependent on circadian timing, and its secretion pattern is a key output of the biological clock. Conventional oral formulations have short half-lives and fail to maintain therapeutic concentrations throughout the night, limiting their efficacy for sleep regulation 4,81. Current sustained-release technologies improve pharmacokinetic profiles by extending systemic exposure, yet even these constant-rate formulations cannot reproduce the physiological nocturnal peak characteristic of endogenous melatonin secretion. In contrast, truly chronotherapeutic systems, which incorporate defined lag times, pulsatile release, or closed-loop feedback, remain largely at the conceptual or early developmental stage. Realizing such systems will require the integration of smart responsive materials capable of sensing and responding to specific physiological triggers 112 (e.g., pH, redox potential, enzyme activity) to achieve site- or time-specific release. Advanced manufacturing technologies such as 3D printing enable fabrication of dosage forms with precisely defined lag times and pulsed or multiphasic release profiles, allowing programmable release kinetics. Further development should include incorporation of biosensing elements to construct closed-loop systems that modulate drug release in real time based on circulating melatonin levels or other physiological biomarkers. Leveraging pharmacogenomics and circadian phenotype data will permit individualization of release profiles and enable personalized chronotherapy. For clinical scenarios such as jet lag, shift-work disorder or circadian rhythm sleep-wake disorders, customized delivery systems with accurate timing control have the potential to align therapeutic interventions with individual circadian patterns.

### 5.4 Regulatory Frameworks and Standardized Assays

Rapid advances in nanotechnology have driven innovation and commercialization in drug delivery 181, but international regulatory frameworks specific to nanomedicines remain under development; the absence of unified global standards is a pervasive challenge 182, 183.

Regulatory difficulties focus on establishing precise and internationally accepted definitions for nanomaterials and nanomedicines, developing standardized characterization methods for complex formulations (e.g., hybrid systems, surface-functionalized particles, multi-component assemblies), validating *in vitro* release tests predictive of *in vivo* performance, and clarifying requirements for clinical traceability, batch documentation and post-marketing surveillance 184. In response to these challenges, regulatory agencies such as the U.S. FDA and the European Medicines Agency (EMA) have published guidance documents for nanotechnology applications and reflection papers on nanomedicines 185, 186. However, they currently rely on existing pharmaceutical frameworks while encouraging case-by-case assessments founded on Quality by Design (QbD) principles 186, 187.

To advance nanomelatonin products toward clinical use, close collaboration between academic researchers and regulators is required to establish QbD-based evaluation frameworks, define critical quality attributes such as particle size, surface charge, drug loading and release behavior, and develop corresponding standardized analytical methods. All excipients used in nanomedicines must meet "generally recognized as safe" criteria, and their altered physicochemical properties at the nanoscale should be evaluated rigorously 188.

## 6. Conclusion

Research on advanced melatonin delivery systems is at a pivotal stage between proof-of-concept and clinical translation. Although optimal dosing, timing and individualized treatment strategies for melatonin remain under investigation, nanotechnology provides powerful engineering approaches to realize its therapeutic potential. Future progress will depend on integrating multiple emerging disciplines: precision manufacturing technologies including microfluidics and 3D printing; biomimetic design principles inspired by endogenous transport mechanisms; smart responsive materials enabling environment-sensing drug release; and chronotherapeutic strategies that synchronize interventions with biological rhythms. Concurrently, a robust regulatory science framework is essential to ensure the safety, quality and efficacy of these advanced formulations. Through multidisciplinary coordination, next-generation melatonin nanomedicines may evolve from dietary supplements into advanced therapeutic platforms for complex neurological disorders, metabolic diseases and cancer, ultimately shifting from substitution of endogenous deficiency toward restoration of physiological homeostasis.

## Figures and Tables

**Table 1:** Summary of Melatonin Nanocarriers: Types, Advantages, and Limitations

Carrier Type	Formulation Material	Key Advantages	Major Limitations	Reference
Liposomes	Phospholipids, Cholesterol	High biocompatibility; mimics cell membranes; easy to functionalize.	Poor stability; low loading for hydrophobic drugs; rapid leakage.	37, 40, 41, 64
SLNs	Solid lipids (e.g., Stearic acid), Surfactants	Controlled release; avoids organic solvents; large-scale production possible.	Drug expulsion during storage due to crystal transition; limited loading space.	54, 58, 59, 62, 63, 67
NLCs	Solid lipids + Liquid oils (e.g., Oleic acid)	High drug loading; improved stability; minimal drug expulsion (imperfect lattice).	Complex manufacturing compared to SLNs; potential particle growth.	59, 60, 61, 63
PLGA nanoparticles	PLGA	Excellent sustained release (weeks/months); biodegradable; FDA-approved.	Use of organic solvents; acidic degradation products may cause local inflammation.	78, 79, 82, 85, 86
Chitosan nanoparticles	Chitosan (Natural polysaccharide)	Mucoadhesive (positive charge); opens tight junctions; excellent for intranasal delivery.	High sensitivity to pH/ionic strength; low mechanical strength.	16, 94, 96, 97
Cyclodextrins	Cyclic oligosaccharides (α, β, γ-CDs)	Enhances solubility of melatonin; improves chemical stability; fast absorption.	Limited controlled-release capability; primarily suitable for solubility enhancement rather than sustained delivery.	102, 104
Hydrogels	Carbopol, Chitosan, Alginate	High water content; prolonged local residence time; tunable viscosity.	High initial burst release; difficult to achieve precise dose control.	76, 112

**Table 2:** Comparative Performance of Nanocarrier Platforms in Overcoming Specific Biological Barriers for Melatonin Delivery

Barrier Challenge	Clinical Need	Most Suitable Carrier Platforms	Mechanism of Action	Limitations/Translational Hurdles	Reference
Hepatic First-Pass Metabolism	Improve oral bioavailability; avoid systemic clearance	PLGA nanoparticles (oral sustained release); Chitosan-based nanocarriers (colon-targeted oral delivery); Microneedles (transdermal bypass)	Polymer matrix protection and sustained release in GI tract (PLGA); pH-responsive colon targeting (Chitosan); Physical stratum corneum penetration creating transient microchannels (Microneedles)	PLGA acidic degradation may affect drug stability; Lymphatic transport efficiency is dose-dependent; Microneedle fabrication cost and skin irritation potential	81, 136, 148
BBB	Neuroprotection (AD, PD); Glioma therapy	Chitosan-based nanocarriers (intranasal); RVG-peptide-modified Liposomes	Paracellular tight junction opening via ZO-1 modulation (Chitosan); Receptor-mediated transcytosis via lactoferrin receptors (Liposomes)	Intranasal dose volume limited by nasal cavity capacity; IV immunogenicity and accelerated blood clearance of PEGylated liposomes	93, 161
Skin Barrier	Sustained overnight sleep maintenance; Local skin effects	Ethosomes; Silk Fibroin Microneedles	Lipid bilayer fluidization and disruption of stratum corneum lipid organization (Ethosomes); Microchannel creation bypassing the skin barrier (Microneedles)	Ethanol-induced skin irritation potential; Microneedle mechanical strength and large-scale fabrication challenges	46, 47, 147
Ocular Clearance	Glaucoma; Retinal protection	Cationic SLNs; In situ Gels	Electrostatic adhesion to negatively charged corneal mucins (Cationic SLNs); Viscosity increase and phase transition upon exposure to tear fluid (In situ Gels)	Potential for transient blurred vision; Shorter residence time compared to invasive implants	57, 158

**Table 3:** Preclinical Studies of Melatonin-Loaded Nanocarriers in Disease Models

Disease Model	Delivery System	Route	Research Model	Key Outcome (Therapeutic Effect)	Reference
Alzheimer's (AD)	Chitosan-coated NLCs	Intranasal	Murine AD model	Crossed BBB; reduced Aβ plaque accumulation and oxidative stress.	126
Parkinson's (PD)	PLGA Nanoparticles	Oral	PD cell model (MPP⁺-induced)	Sustained release; protected dopaminergic neurons in Substantia Nigra.	167
Wound Healing	Chitosan/Collagen Film	Topical	Rodent skin wound model	Accelerated re-epithelialization, reduced scar formation.	96
Colitis	Targeted Nano-carriers	Oral	Murine colitis model	Specific accumulation in inflamed colon; restored gut microbiota.	136
Liver Fibrosis	PLGA-PEG Micelles	Intravenous	CCl₄-induced rat liver injury model	Reduced hepatic stellate cell activation and collagen deposition.	168
Spinal Cord Injury	In-situ Hydrogel	Intrathecal	Rat spinal cord compression model	Sustained release at lesion site; promoted locomotor recovery.	169
Myocardial Infarction	Mel@ADSC NVs (Melatonin-engineered adipose-derived stem cell nanovesicles)	Intravenous	Murine myocardial infarction model	Reduced apoptosis (42.59%→13.88%), alleviated ROS, promoted angiogenesis, improved mitochondrial function.	170
Retinal Degeneration	Niosomes (Vesicular)	Topical (Eye)	Rabbit retinal degeneration model	Enhanced corneal permeation; reduced retinal oxidative damage.	171
Depression	Polymeric Micelles	Intranasal	Murine sleep deprivation model	Rapid brain delivery, restored circadian rhythm/ rapid eye movement sleep, ameliorated depression.	172
Radiation-Induced Lung Injury (RILI)	Melatonin-loaded PLGA nanoparticles	Intratracheal instillation	SD rat RILI model (15 Gy irradiation)	Alleviated radiation-induced lung inflammation and fibrosis via the miR-21/TGF-β1/Smad3 pathway.	173
Osteosarcoma	PLGA Micro/Nanoparticles	Local/*In vitro*	MG-63 osteosarcoma cell model	Sustained release (~70% in 40d); inhibited osteosarcoma cell growth; adjuvant to chemotherapy.	81
Subarachnoid Hemorrhage (SAH)	Melatonin-loaded PLGA injectable nanosuspension	Intravenous	Rat SAH model	Sustained release (120h), crossed BBB, reduced mortality, brain edema, antioxidant.	174
Osteoarthritis (OA)	Hyaluronic acid-Cyclodextrin Melatonin Delivery System	Intra-articular	Rat OA model	Sustained release; restored chondrocyte mitochondrial function; promoted cartilage extracellular matrix synthesis.	103
Glaucoma (Ocular Hypertension)	Melatonin-loaded cationic SLNs	Topical (Ocular)	Albino rabbit ocular hypertension model	Reduced IOP (max -7 mmHg, p<0.01); 24h duration; good ocular tolerance.	57
Breast Cancer	Melatonin-loaded lecithin/chitosan nanoparticles	Intravenous / Oral	4T1 metastatic breast cancer cell model; BALB/c mouse tumor model	High encapsulation (31%), controlled release, selective cytotoxicity (SI=13.33), low systemic toxicity.	175
Sleep Deprivation	Polymeric Micelles	Intranasal	Murine sleep deprivation model	Rapid brain delivery; restored circadian rhythm and rapid eye movement sleep.	127
Sleep Maintenance / Insomnia	SLNs	Oral / Transdermal	Healthy human subjects	Oral MT-SLN: significantly higher AUC (∼2×) and prolonged elimination half-life (∼93 vs 48 min); Transdermal MT-SLN: sustained plasma levels (>50 pg/mL) for at least 24 h	56
Circadian Rhythm Disruption / Insomnia	Silk Fibroin Microneedles	Transdermal	Rat insomnia model	Sustained release for >11 h (>85% cumulative release); maintained therapeutic plasma levels for ∼8 h; improved sleep architecture and reduced anxiety-like behavior	147
Ischemic Stroke	PEGylated Liposomes	Intravenous	Murine middle cerebral artery occlusion stroke model	Extended half-life; minimized brain infarct volume via anti-apoptosis.	176
Glioblastoma	Polycaprolactone melatonin nanoparticles; Melatonin-pre-treated mesenchymal stem cells	Intranasal	Rat *in vivo* model; U87MG glioblastoma cell line; MRC-5 non-tumor cell line	35× solubility, high cytotoxicity to U87MG (IC_50_ 2500× lower), non-toxic to MRC-5, direct nose-to-brain transport, high targeting index.	124

Notes: Mel@ADSC NVs: melatonin-engineered adipose-derived stem cell nanovesicles

## Abbreviations

Aβ: amyloid beta-protein
ABCB1: ATP-binding cassette subfamily B member 1
AD: Alzheimer's disease
ADSC NVs: adipose-derived stem cell nanovesicles
AFMK: N¹-acetyl-N²-formyl-5-methoxykynuramine
AMK: N¹-acetyl-5-methoxykynuramine
AUC: area under the curve
BBB: blood-brain barrier
BMP-2: bone morphogenetic protein-2
CDs: cyclodextrins
CNS: central nervous system
CYP: cytochrome P450
CYP1A2: cytochrome P450 1A2
DDS: drug delivery systems
EMA: European Medicines Agency
FDA: Food and Drug Administration
GRAS: Generally Recognized as Safe
IC₅₀: half-maximal inhibitory concentration
IOP: intraocular pressure
IR: immediate-release
LNCs: lipidic nanocapsules
MT1: melatonin receptor 1
MT2: melatonin receptor 2
NLCs: nanostructured lipid carriers
OA: osteoarthritis
PD: Parkinson's disease
PEG: polyethylene glycol
P-gp: P-glycoprotein
PLGA: poly(lactic-co-glycolic acid)
PR: prolonged-release
QbD: Quality by Design
RILI: radiation-induced lung injury
RORα: retinoic acid receptor-related orphan receptor alpha
ROS: reactive oxygen species
RNS: reactive nitrogen species
RVG: rabies virus glycoprotein
SAH: subarachnoid hemorrhage
SLNs: solid lipid nanoparticles
SNEDDS: self-nanoemulsifying drug delivery systems
ZO-1: zonula occludens-1

## References

[1] Reiter RJ, Rosales-Corral S, Tan DX (2017). Melatonin as a mitochondria-targeted antioxidant: one of evolution's best ideas. Cell Mol Life Sci.

[2] Manchester LC, Poeggeler B, Alvarez FL (1995). Melatonin immunoreactivity in the photosynthetic prokaryote Rhodospirillum rubrum: implications for an ancient antioxidant system. Cell Mol Biol Res.

[3] Lerner AB, Case JD, Takahashi Y (1958). Isolation of melatonin, the pineal gland factor that lightens melanocytes. J Am Chem Soc.

[4] Chuffa LGA, Seiva FRF, Novais AA (2021). Melatonin-loaded nanocarriers: new horizons for therapeutic applications. Molecules.

[5] Acuña-Castroviejo D, Escames G, Venegas C (2014). Extrapineal melatonin: sources, regulation, and potential functions. Cell Mol Life Sci.

[6] Amaral FGD, Cipolla-Neto J (2018). A brief review about melatonin, a pineal hormone. Arch Endocrinol Metab.

[7] Pandiperumal S, Trakht I, Srinivasan V (2008). Physiological effects of melatonin: role of melatonin receptors and signal transduction pathways. Prog Neurobiol.

[8] Galano A, Tan DX, Reiter RJ (2013). On the free radical scavenging activities of melatonin's metabolites, AFMK and AMK. J Pineal Res.

[9] Acuna-Castroviejo D, Martin M, Macias M (2001). Melatonin, mitochondria and cellular bioenergetics. J Pineal Res.

[10] Cardinali DP (2019). Melatonin: clinical perspectives in neurodegeneration. Front Endocrinol (Lausanne).

[11] Cho JH, Bhutani S, Kim CH, Irwin MR (2021). Anti-inflammatory effects of melatonin: a systematic review and meta-analysis of clinical trials. Brain Behav Immun.

[12] Yi YJ, Tang H, Pi PL (2024). Melatonin in cancer biology: pathways, derivatives, and the promise of targeted delivery. Drug Metab Rev.

[13] Guan Q, Wang Z, Cao J, Dong Y, Chen Y (2021). Mechanisms of melatonin in obesity: a review. Int J Mol Sci.

[14] Dalgic AD, Atila D, Tezcaner A (2023). Diatom silica frustules-doped fibers for controlled release of melatonin for bone regeneration. Eur Polym J.

[15] Lalanne S (2021). Melatonin: from pharmacokinetics to clinical use in autism spectrum disorder. Int J Mol Sci.

[16] Hafner A, Lovrić J, Voinovich D, Filipović-Grcić J (2009). Melatonin-loaded lecithin/chitosan nanoparticles: physicochemical characterisation and permeability through Caco-2 cell monolayers. Int J Pharm.

[17] Canizo BV, Jofré MF, Mammana SB (2024). Stability of melatonin in eutectic systems: new avenues in therapeutic product development. J Ionic Liquids.

[18] Johns JR, Chenboonthai C, Johns NP, Saengkrasat A, Kuketpitakwong R, Porasupatana S (2012). An intravenous injection of melatonin: formulation, stability, pharmacokinetics and pharmacodynamics. J Asian Assoc Sch Pharm.

[19] Harpsøe NG, Andersen LPH, Gögenur I, Rosenberg J (2015). Clinical pharmacokinetics of melatonin: a systematic review. Eur J Clin Pharmacol.

[20] Facciolá G, Hidestrand M, von Bahr C, Tybring G (2001). Cytochrome P450 isoforms involved in melatonin metabolism in human liver microsomes. Eur J Clin Pharmacol.

[21] Tordjman S, Chokron S, Delorme R (2017). Melatonin: pharmacology, functions and therapeutic benefits. Curr Neuropharmacol.

[22] Andersen LPH, Gögenur I, Rosenberg J, Reiter RJ (2016). Pharmacokinetics of melatonin: the missing link in clinical efficacy?. Clin Pharmacokinet.

[23] Reiter RJ, Tan DX, Kim SJ, Cruz MH (2014). Delivery of pineal melatonin to the brain and SCN: role of canaliculi, cerebrospinal fluid, tanycytes and Virchow-Robin perivascular spaces. Brain Struct Funct.

[24] Luo F, Deng Y, Angelov B, Angelova A (2025). Melatonin and the nervous system: nanomedicine perspectives. Biomater Sci.

[25] Blanchette M, Bajc K, Gastfriend BD (2025). Regional heterogeneity of the blood-brain barrier. Nat Commun.

[26] Roberts DJ, Goralski KB (2008). A critical overview of the influence of inflammation and infection on P-glycoprotein expression and activity in the brain. Expert Opin Drug Metab Toxicol.

[27] Hartz AM, Bauer B, Fricker G, Miller DS (2006). Rapid modulation of P-glycoprotein-mediated transport at the blood-brain barrier by tumor necrosis factor-alpha and lipopolysaccharide. Mol Pharmacol.

[28] Lin JH, Yamazaki M (2003). Role of P-glycoprotein in pharmacokinetics: clinical implications. Clin Pharmacokinet.

[29] Saiz-Rodríguez M, Ochoa D, Belmonte C (2019). Polymorphisms in CYP1A2, CYP2C9 and ABCB1 affect agomelatine pharmacokinetics. J Psychopharmacol.

[30] Phatale V, Vaiphei KK, Jha S (2022). Overcoming skin barriers through advanced transdermal drug delivery approaches. J Control Release.

[31] Boutin JA, Jockers R (2021). Melatonin controversies, an update. J Pineal Res.

[32] Tran HTT, Tran PHL, Lee B-J (2009). New findings on melatonin absorption and alterations by pharmaceutical excipients using the Ussing chamber technique with mounted rat gastrointestinal segments. Int J Pharm.

[33] Ambrogio MW, Frasconi M, Yilmaz MD, Chen X (2013). New methods for improved characterization of silica nanoparticle-based drug delivery systems. Langmuir.

[34] Goel H, Kalra V, Verma SK (2022). Convolutions in the rendition of nose to brain therapeutics from bench to bedside: feats & fallacies. J Control Release.

[35] Fan Y, Chen M, Zhang J (2018). Updated progress of nanocarrier-based intranasal drug delivery systems for treatment of brain diseases. Crit Rev Ther Drug Carrier Syst.

[36] Hubbell JA, Langer R (2013). Translating materials design to the clinic. Nat Mater.

[37] Allen TM, Cullis PR (2013). Liposomal drug delivery systems: from concept to clinical applications. Adv Drug Deliv Rev.

[38] Liu C, Ewert KK, Wang N (2019). A multifunctional lipid that forms contrast-agent liposomes with dual-control release capabilities for precise MRI-guided drug delivery. Biomaterials.

[39] Molska A, Nyman AKG, Sofias AM (2020). In vitro and in vivo evaluation of organic solvent-free injectable melatonin nanoformulations. Eur J Pharm Biopharm.

[40] Ishida T, Harashima H, Kiwada H (2012). Liposome clearance. Biosci Rep.

[41] Kraft JC, Freeling JP, Wang Z, Ho RJY (2014). Emerging research and clinical development trends of liposome and lipid nanoparticle drug delivery systems. J Pharm Sci.

[42] Merino M, Zalba S, Garrido MJ (2018). Immunoliposomes in clinical oncology: state of the art and future perspectives. J Control Release.

[43] Teixeira MC, Carbone C, Souto EB (2017). Beyond liposomes: recent advances on lipid based nanostructures for poorly soluble/poorly permeable drug delivery. Prog Lipid Res.

[44] Fernández-García R, Lalatsa A, Statts L (2020). Transferosomes as nanocarriers for drugs across the skin: quality by design from lab to industrial scale. Int J Pharm.

[45] Cevc G, Blume G (1992). Lipid vesicles penetrate into intact skin owing to the transdermal osmotic gradients and hydration force. Biochim Biophys Acta Biomembr.

[46] Paiva-Santos AC, Silva AL, Guerra C (2021). Ethosomes as nanocarriers for the development of skin delivery formulations. Pharm Res.

[47] Dubey V, Mishra D, Jain N (2007). Melatonin loaded ethanolic liposomes: physicochemical characterization and enhanced transdermal delivery. Eur J Pharm Biopharm.

[48] Jefferies WA, Brandon MR, Hunt SV (1984). Transferrin receptor on endothelium of brain capillaries. Nature.

[49] Thomsen LB, Linemann T, Birkelund S (2019). Evaluation of targeted delivery to the brain using magnetic immunoliposomes and magnetic force. Materials (Basel).

[50] Raju R, Abuwatfa WH, Pitt WG, Husseini GA (2023). Liposomes for the treatment of brain cancer-a review. Pharmaceuticals (Basel).

[51] El Maghraby GM, Barry BW, Williams AC (2008). Liposomes and skin: from drug delivery to model membranes. Eur J Pharm Sci.

[52] Subongkot T, Pamornpathomkul B, Rojanarata T (2014). Investigation of the mechanism of enhanced skin penetration by ultradeformable liposomes. Int J Nanomedicine.

[53] Samimi S, Maghsoudnia N, Eftekhari RB, Dorkoosh F (2019). Lipid-based nanoparticles for drug delivery systems. In: Mohapatra S, Ranjan S, Dasgupta N, Thomas S, Mishra RK, eds. Characterization and Biology of Nanomaterials for Drug Delivery. Amsterdam: Elsevier.

[54] Severino P, Andreani T, Macedo AS (2012). Current state-of-art and new trends on lipid nanoparticles (SLN and NLC) for oral drug delivery. J Drug Deliv.

[55] Tursilli R, Casolari A, Iannuccelli V, Scalia S (2006). Enhancement of melatonin photostability by encapsulation in lipospheres. J Pharm Biomed Anal.

[56] Priano L, Esposti D, Esposti R (2007). Solid lipid nanoparticles incorporating melatonin as new model for sustained oral and transdermal delivery systems. J Nanosci Nanotechnol.

[57] Leonardi A, Bucolo C, Drago F (2015). Cationic solid lipid nanoparticles enhance ocular hypotensive effect of melatonin in rabbit. Int J Pharm.

[58] Xu L, Wang X, Liu Y (2022). Lipid nanoparticles for drug delivery. Adv Nanobiomed Res.

[59] Müller RH, Radtke M, Wissing SA (2002). Nanostructured lipid matrices for improved microencapsulation of drugs. Int J Pharm.

[60] Apostolou M, Assi S, Fatokun AA, Khan I (2021). The effects of solid and liquid lipids on the physicochemical properties of nanostructured lipid carriers. J Pharm Sci.

[61] Hatem S, Nasr M, Moftah NH (2018). Clinical cosmeceutical repurposing of melatonin in androgenic alopecia using nanostructured lipid carriers prepared with antioxidant oils. Expert Opin Drug Deliv.

[62] Mirchandani Y, Patravale VB, Brijesh S (2021). Solid lipid nanoparticles for hydrophilic drugs. J Control Release.

[63] Ghosh S, Tiwari T, Nagaich U, Jain N (2023). A detailed insight into nanostructured lipid carriers: a versatile drug delivery system. Recent Pat Nanotechnol.

[64] Zhang Q, Ou C, Ye S (2017). Construction of nanoscale liposomes loaded with melatonin via supercritical fluid technology. J Microencapsul.

[65] Lombardo D, Kiselev MA (2022). Methods of liposomes preparation: formation and control factors of versatile nanocarriers for biomedical and nanomedicine application. Pharmaceutics.

[66] Elshall AA, Ghoneim AM, Abdel-Mageed HM (2022). Ex vivo permeation parameters and skin deposition of melatonin-loaded microemulsion for treatment of alopecia. Futur J Pharm Sci.

[67] Souto EB, Doktorovová S (2009). Solid lipid nanoparticle formulations: pharmacokinetic and biopharmaceutical aspects in drug delivery. Methods Enzymol.

[68] Simone E, Ding BS, Muzykantov V (2009). Targeted delivery of therapeutics to endothelium. Cell Tissue Res.

[69] Mohamed M, Abu Lila AS, Shimizu T (2019). PEGylated liposomes: immunological responses. Sci Technol Adv Mater.

[70] Thi TTH, Pilkington EH, Nguyen DH (2020). The importance of poly(ethylene glycol) alternatives for overcoming PEG immunogenicity in drug delivery and bioconjugation. Polymers (Basel).

[71] Dai Y, Wang B, Sun Z (2019). Multifunctional theranostic liposomes loaded with a hypoxia-activated prodrug for cascade-activated tumor selective combination therapy. ACS Appl Mater Interfaces.

[72] Bioavailability of Oniria®, a melatonin prolonged-release formulation, versus immediate-release melatonin in healthy volunteers Drugs R D. 2022; 22: 235-243.

[73] Chua HM, Richer NH, Swedrowska M (2016). Dissolution of intact, divided and crushed Circadin tablets: prolonged vs. immediate release of melatonin. Pharmaceutics.

[74] Ritger PL, Peppas NA (1987). A simple equation for description of solute release. II Fickian and anomalous release from swellable devices. J Control Release.

[75] Cheaburu-Yilmaz CN, Atmaca K, Yilmaz O, Orhan H (2024). Development, characterization, and evaluation of potential systemic toxicity of a novel oral melatonin formulation. Pharmaceutics.

[76] Yao Z, Qian Y, Jin Y (2022). Biomimetic multilayer polycaprolactone/sodium alginate hydrogel scaffolds loaded with melatonin facilitate tendon regeneration. Carbohydr Polym.

[77] Ahlawat J, Henriquez G, Narayan M (2018). Enhancing the delivery of chemotherapeutics: role of biodegradable polymeric nanoparticles. Molecules.

[78] Vauthier C, Bouchemal K (2008). Methods for the preparation and manufacture of polymeric nanoparticles. Pharm Res.

[79] Danhier F, Ansorena E, Silva JM (2012). PLGA-based nanoparticles: an overview of biomedical applications. J Control Release.

[80] Rezvantalab S, Drude NI, Moraveji MK (2018). PLGA-based nanoparticles in cancer treatment. Front Pharmacol.

[81] Altındal DÇ, Gümüşderelioğlu M (2016). Melatonin releasing PLGA micro/nanoparticles and their effect on osteosarcoma cells. J Microencapsul.

[82] Jumadi J, Harun WSW, Kadirgama K (2026). A comparative review of polylactic acid and poly(lactic-co-glycolic acid) biomaterials: optimizing drug delivery. BioNanoScience.

[83] Wang Y, Mo Y, Sun Y (2024). Intestinal nanoparticle delivery and cellular response: a review of the bidirectional nanoparticle-cell interplay in mucosa based on physiochemical properties. J Nanobiotechnology.

[84] Fu K, Pack DW, Klibanov AM, Langer R (2000). Visual evidence of acidic environment within degrading poly(lactic-co-glycolic acid) (PLGA) microspheres. Pharm Res.

[85] Ding AG, Shenderova A, Schwendeman SP (2006). Prediction of microclimate pH in poly(lactic-co-glycolic acid) films. J Am Chem Soc.

[86] Liu Y, Ghassemi AH, Hennink WE, Schwendeman SP (2012). The microclimate pH in poly(D,L-lactide-co-hydroxymethyl glycolide) microspheres during biodegradation. Biomaterials.

[87] Zhu G, Schwendeman SP (2000). Stabilization of proteins encapsulated in cylindrical poly(lactide-co-glycolide) implants: mechanism of stabilization by basic additives. Pharm Res.

[88] Kesharwani P, Kumar V, Goh KW (2025). PEGylated PLGA nanoparticles: unlocking advanced strategies for cancer therapy. Mol Cancer.

[89] Son J, Yang SM, Yi G (2018). Folate-modified PLGA nanoparticles for tumor-targeted delivery of pheophorbide a in vivo. Biochem Biophys Res Commun.

[90] Das U, Kapoor DU, Singh S, Prajapati BG (2024). Unveiling the potential of chitosan-coated lipid nanoparticles in drug delivery for management of critical illness: a review. Z Naturforsch C J Biosci.

[91] Wang Z, Yu F, Hu F (2024). Functional chitosan and its derivative-related drug delivery systems for nano-therapy: recent advances. Pharmaceutics.

[92] Vllasaliu D, Exposito-Harris R, Heras A (2010). Tight junction modulation by chitosan nanoparticles: comparison with chitosan solution. Int J Pharm.

[93] Wang Y, Chen L, Wang Y (2023). Marine biomaterials in biomedical nano/micro-systems. J Nanobiotechnology.

[94] Casettari L, Illum L (2014). Chitosan in nasal delivery systems for therapeutic drugs. J Control Release.

[95] Yadav SK, Srivastava AK, Dev A (2017). Nanomelatonin triggers superior anticancer functionality in a human malignant glioblastoma cell line. Nanotechnology.

[96] Blažević F, Milekić T, Romić MD (2016). Nanoparticle-mediated interplay of chitosan and melatonin for improved wound epithelialisation. Carbohydr Polym.

[97] Hafner A, Dürrigl M, Pepić I, Filipović-Grcić J (2011). Short- and long-term stability of lyophilised melatonin-loaded lecithin/chitosan nanoparticles. Pharm Bull.

[98] Thanou MM, Kotzé AF, Scharringhausen T (2000). Effect of degree of quaternization of N-trimethyl chitosan chloride for enhanced transport of hydrophilic compounds across intestinal Caco-2 cell monolayers. J Control Release.

[99] Urbaniak T, García-Briones GS, Zhigunov A (2022). Quaternized chitosan/heparin polyelectrolyte multilayer films for protein delivery. Biomacromolecules.

[100] Jarrar H, Çetin Altındal D, Gümüşderelioğlu M (2021). Effect of melatonin/BMP-2 co-delivery scaffolds on the osteoclast activity. J Mater Sci Mater Med.

[101] Jarrar H, Çetin Altındal D, Gümüşderelioğlu M (2021). Scaffold-based osteogenic dual delivery system with melatonin and BMP-2 releasing PLGA microparticles. Int J Pharm.

[102] Santhamoorthy M, Asaithambi P, Ramkumar V, Elangovan N, Perumal I, Kim SC (2025). A review on the recent advancements of polymer-modified mesoporous silica nanoparticles for drug delivery under stimuli-trigger. Polymers (Basel).

[103] Zhang Y, Hou M, Liu Y (2022). Recharge of chondrocyte mitochondria by sustained release of melatonin protects cartilage matrix homeostasis in osteoarthritis. J Pineal Res.

[104] Hoti G, Ferrero R, Caldera F (2023). A comparison between the molecularly imprinted and non-molecularly imprinted cyclodextrin-based nanosponges for the transdermal delivery of melatonin. Polymers (Basel).

[105] Achilleos DS, Vamvakaki M (2010). End-grafted polymer chains onto inorganic nano-objects. Materials (Basel).

[106] Gann JP, Yan M (2008). A versatile method for grafting polymers on nanoparticles. Langmuir.

[107] Bitar A, Ahmad NM, Fessi H, Elaissari A (2012). Silica-based nanoparticles for biomedical applications. Drug Discov Today.

[108] El-Megharbel SM, Almalki ASA, Hamza RZ (2018). Synthesis and suggestion of a new nanometric gold(III) melatonin drug complex: an interesting model for testicular protection. Future Med Chem.

[109] Gurunathan S, Jeyaraj M, Kang M-H, Kim J-H (2020). Melatonin enhances palladium-nanoparticle-induced cytotoxicity and apoptosis in human lung epithelial adenocarcinoma cells A549 and H1229. Antioxidants (Basel).

[110] Moody AS, Sharma B (2018). Multi-metal, multi-wavelength surface-enhanced Raman spectroscopy detection of neurotransmitters. ACS Chem Neurosci.

[111] Liu C, Li Y, Liu X (2025). Carbon dots-based drug delivery for bone regeneration. Front Bioeng Biotechnol.

[112] Wu Y, Wang Y, Long L (2022). A spatiotemporal release platform based on pH/ROS stimuli-responsive hydrogel in wound repairing. J Control Release.

[113] Sonvico F, Clementino A, Buttini F (2018). Surface-modified nanocarriers for nose-to-brain delivery: from bioadhesion to targeting. Pharmaceutics.

[114] Wu D, Chen Q, Chen X (2023). The blood-brain barrier: structure, regulation and drug delivery. Signal Transduct Target Ther.

[115] Marcello E, Chiono V (2023). Biomaterials-enhanced intranasal delivery of drugs as a direct route for brain targeting. Int J Mol Sci.

[116] Ruigrok MJR, de Lange ECM (2015). Emerging insights for translational pharmacokinetic and pharmacokinetic-pharmacodynamic studies: towards prediction of nose-to-brain transport in humans. AAPS J.

[117] Maaz A, Blagbrough IS, De Bank PA (2021). In vitro evaluation of nasal aerosol depositions: an insight for direct nose to brain drug delivery. Pharmaceutics.

[118] Agrawal M, Saraf S, Saraf S (2018). Nose-to-brain drug delivery: an update on clinical challenges and progress towards approval of anti-Alzheimer drugs. J Control Release.

[119] Shadab M, Bhattmisra SK, Zeeshan F (2018). Nano-carrier enabled drug delivery systems for nose to brain targeting for the treatment of neurodegenerative disorders. J Drug Deliv Sci Technol.

[120] Feng Y, He H, Li F (2018). An update on the role of nanovehicles in nose-to-brain drug delivery. Drug Discov Today.

[121] Samaridou E, Alonso MJ (2018). Nose-to-brain peptide delivery - the potential of nanotechnology. Bioorg Med Chem.

[122] Esfandyari-Manesh M, Mohammadi A, Atyabi F (2016). Specific targeting delivery to MUC1 overexpressing tumors by albumin-chitosan nanoparticles conjugated to DNA aptamer. Int J Pharm.

[123] Piazzini V, Landucci E, D'Ambrosio M (2019). Chitosan coated human serum albumin nanoparticles: a promising strategy for nose-to-brain drug delivery. Int J Biol Macromol.

[124] De Oliveira Junior ER, Nascimento TL, Salomão MA (2019). Increased nose-to-brain delivery of melatonin mediated by polycaprolactone nanoparticles for the treatment of glioblastoma. Pharm Res.

[125] Bseiso EA, AbdEl-Aal SA, Nasr M (2022). Nose to brain delivery of melatonin lipidic nanocapsules as a promising post-ischemic neuroprotective therapeutic modality. Drug Deliv.

[126] Srivastava AK, Roy Choudhury S, Karmakar S (2020). Near-infrared responsive dopamine/melatonin-derived nanocomposites abrogating in situ amyloid β nucleation, propagation, and ameliorate neuronal functions. ACS Appl Mater Interfaces.

[127] Priprem A, Johns JR, Limsitthichaikoon S (2017). Intranasal melatonin nanoniosomes: pharmacokinetic, pharmacodynamics and toxicity studies. Ther Deliv.

[128] Keller L-A, Merkel O, Popp A (2021). Intranasal drug delivery: opportunities and toxicologic challenges during drug development. Drug Deliv Transl Res.

[129] Wang H, Li J, Jin J (2022). Enhanced efficiency of melatonin by stepwise-targeting strategy for acute lung injury. Front Bioeng Biotechnol.

[130] Ryan R, Leslie MN, He P (2025). Intranasal and inhaled delivery systems for targeting circadian dysfunction in neurodegenerative disorders, perspective and future outlook. Adv Drug Deliv Rev.

[131] Kumar R, Mehta P, Shankar KR (2022). Nanotechnology-assisted metered-dose inhalers (MDIs) for high-performance pulmonary drug delivery applications. Pharm Res.

[132] Kolli AR, Kuczaj AK, Calvino-Martin F, Hoeng J (2024). Simulated pharmacokinetics of inhaled caffeine and melatonin from existing products indicate the lack of dosimetric considerations. Food Chem Toxicol.

[133] Ling J, Mangal S, Park H (2019). Simultaneous particle size reduction and homogeneous mixing to produce combinational powder formulations for inhalation by the single-step co-jet milling. J Pharm Sci.

[134] Rongve A, Boeve BF, Aarsland D (2010). Frequency and correlates of caregiver-reported sleep disturbances in a sample of persons with early dementia. J Am Geriatr Soc.

[135] Bandyopadhyay D, Biswas K, Bandyopadhyay U (2000). Melatonin protects against stress-induced gastric lesions by scavenging the hydroxyl radical. J Pineal Res.

[136] Mohanbhai SJ, Sardoiwala MN, Gupta S (2022). Colon targeted chitosan-melatonin nanotherapy for preclinical inflammatory bowel disease. Biomater Adv.

[137] Butkevych T, Polova Z, Savchenko S (2023). Development of the composition and technology of an orodispersible film with melatonin and magnesium citrate. Ukr Sci Med Youth J.

[138] Yen YW, Lee YL, Yu LY (2023). Fucoidan/chitosan layered PLGA nanoparticles with melatonin loading for inducing intestinal absorption and addressing triple-negative breast cancer progression. Int J Biol Macromol.

[139] Jarak I, Silva I, Domingues C (2022). Nanofiber carriers of therapeutic load: current trends. Int J Mol Sci.

[140] Shahrokh S, Namazi N, Abbasinazari M, Abazarikia A, Sadeghi A, Mahboubi A (2025). Assessment of the efficacy and safety of sublingual melatonin on symptom severity, quality of life, and sleep disorders in patients with irritable bowel syndrome. Iran J Pharm Res.

[141] Shahgordi S, Nosratzadeh N, Kaffash E (2025). Development of sublingual melatonin nanofibers in enhancing the anticancer effects of doxorubicin, in vitro and in vivo studies. J Drug Deliv Sci Technol.

[142] Speer I, Preis M, Lenhart V (2018). Prolonged drug release properties for orodispersible films by combining hot-melt extrusion and solvent casting methods. Eur J Pharm Biopharm.

[143] Speer I, Lenhart V, Preis M (2019). Prolonged release from orodispersible films by incorporation of diclofenac-loaded micropellets. Int J Pharm.

[144] Paudel KS (2010). Challenges and opportunities in dermal/transdermal delivery. Ther Deliv.

[145] Jiang X, Zhao H, Li W (2022). Microneedle-mediated transdermal delivery of drug-carrying nanoparticles. Front Bioeng Biotechnol.

[146] Kundu B, Wang X, Kim HJ (2013). Silk fibroin biomaterials for tissue regenerations. Adv Drug Deliv Rev.

[147] Li X, Chen Y, Shao H (2025). Silk fibroin microneedles loaded with melatonin for circadian rhythm regulation. Int J Biol Macromol.

[148] Qi Z, Cao J, Tao X (2021). Silk fibroin microneedle patches for the treatment of insomnia. Pharmaceutics.

[149] Lyu S, Dong Z, Xu X (2023). Going below and beyond the surface: microneedle structure, materials, drugs, fabrication, and applications for wound healing and tissue regeneration. Bioact Mater.

[150] Abd-El-Azim H, Tekko IA, Ali A (2022). Hollow microneedle assisted intradermal delivery of hypericin lipid nanocapsules with light enabled photodynamic therapy against skin cancer. J Control Release.

[151] Dawud H, Abu Ammar A (2023). Rapidly dissolving microneedles for the delivery of steroid-loaded nanoparticles intended for the treatment of inflammatory skin diseases. Pharmaceutics.

[152] Ingrole RSJ, Gill HS (2019). Microneedle coating methods: a review with a perspective. J Pharmacol Exp Ther.

[153] Chauhan S, Venuganti VVK (2025). Fabrication of 3D printed microneedle patch for the simultaneous delivery and detection of melatonin from interstitial fluid. AAPS PharmSciTech.

[154] Aykora D (2025). 3D bioprinting strategies for melatonin-loaded polymers in bone tissue engineering. Macromol Mater Eng.

[155] Biswas AA, Dhondale MR, Agrawal AK (2024). Advancements in microneedle fabrication techniques: artificial intelligence assisted 3D-printing technology. Drug Deliv Transl Res.

[156] Musumeci T, Bucolo C, Carbone C (2013). Polymeric nanoparticles augment the ocular hypotensive effect of melatonin in rabbits. Int J Pharm.

[157] Romeo A, Bonaccorso A, Carbone C (2022). Melatonin loaded hybrid nanomedicine: DoE approach, optimization and in vitro study on diabetic retinopathy model. Int J Pharm.

[158] Romeo A, Kazsoki A, Musumeci T, Zelkó R (2024). A clinical, pharmacological, and formulation evaluation of melatonin in the treatment of ocular disorders-a systematic review. Int J Mol Sci.

[159] Wang D, Jiang Q, Dong Z (2023). Nanocarriers transport across the gastrointestinal barriers: the contribution to oral bioavailability via blood circulation and lymphatic pathway. Adv Drug Deliv Rev.

[160] Nguyen HX, Banga AK (2025). Advanced transdermal drug delivery system: a comprehensive review of microneedle technologies, novel designs, diverse applications, and critical challenges. Int J Pharm.

[161] Wang X, Chen J, Xia J (2026). Brain-targeted RVG-liposomal melatonin ameliorates manganese neurotoxicity by enhancing neurogenesis and modulating systemic amino acid profiles. J Pineal Res.

[162] Zhi K, Raji B, Nookala AR (2021). PLGA nanoparticle-based formulations to cross the blood-brain barrier for drug delivery: from R&D to cGMP. Pharmaceutics.

[163] Müller RH, Radtke M, Wissing SA (2002). Solid lipid nanoparticles (SLN) and nanostructured lipid carriers (NLC) in cosmetic and dermatological preparations. Adv Drug Deliv Rev.

[164] Hua S, de Matos MBC, Metselaar JM, Storm G (2018). Current trends and challenges in the clinical translation of nanoparticulate nanomedicines: pathways for translational development and commercialization. Front Pharmacol.

[165] Circadin 2 mg prolonged release tablets - Summary of Product Characteristics. European Medicines Agency.

[166] Slenyto 1 mg/5 mg prolonged release tablets - Summary of Product Characteristics. European Medicines Agency.

[167] Jung YJ, Choi H, Oh E (2022). Melatonin attenuates MPP⁺-induced apoptosis via heat shock protein in a Parkinson's disease model. Biochem Biophys Res Commun.

[168] Farid A, Michael V, Safwat G (2023). Melatonin loaded poly(lactic-co-glycolic acid) (PLGA) nanoparticles reduce inflammation, inhibit apoptosis and protect rat's liver from the hazardous effects of CCl4. Sci Rep.

[169] Yao M, Pu PM, Li ZY (2023). Melatonin restores endoplasmic reticulum homeostasis to protect injured neurons in a rat model of chronic cervical cord compression. J Pineal Res.

[170] Zhang Y, Yang N, Huang X (2022). Melatonin engineered adipose-derived biomimetic nanovesicles regulate mitochondrial functions and promote myocardial repair in myocardial infarction. Front Cardiovasc Med.

[171] Bessone CDV, Martinez SM, Luna JD (2020). Neuroprotective effect of melatonin loaded in ethylcellulose nanoparticles applied topically in a retinal degeneration model in rabbits. Exp Eye Res.

[172] Tonon AC, Pilz LK, Markus RP, Hidalgo MP, Elisabetsky E (2021). Melatonin and depression: a translational perspective from animal models to clinical studies. Front Psychiatry.

[173] Wang S, Li J, He Y (2021). Protective effect of melatonin entrapped PLGA nanoparticles on radiation-induced lung injury through the miR-21/TGF-β1/Smad3 pathway. Int J Pharm.

[174] Qian N (2025). Assessment and evaluation of melatonin loaded PLGA injectable nanosuspension for the treatment of subarachnoid hemorrhage: preclinical study. Pak J Pharm Sci.

[175] Franco PIR, do Carmo Neto JR, Braga YLL (2025). Melatonin-loaded lecithin and chitosan nanoparticles are cytotoxic to 4T1 breast cancer cells and safe in a BALB/c mouse model. Int J Biol Macromol.

[176] Sohail S, Shah FA, Zaman SU (2023). Melatonin delivered in solid lipid nanoparticles ameliorated its neuroprotective effects in cerebral ischemia. Heliyon.

[177] Ongoren B, Kara A, Casettari L (2024). Leveraging 3D-printed microfluidic micromixers for the continuous manufacture of melatonin loaded SNEDDS with enhanced antioxidant activity and skin permeability. Int J Pharm.

[178] Arendt J, Skene DJ (2005). Melatonin as a chronobiotic. Sleep Med Rev.

[179] Buscemi N, Vandermeer B, Hooton N (2005). The efficacy and safety of exogenous melatonin for primary sleep disorders a meta-analysis. J Gen Intern Med.

[180] Mirza-Aghazadeh-Attari M, Mihanfar A, Yousefi B, Majidinia M (2022). Nanotechnology-based advances in the efficient delivery of melatonin. Cancer Cell Int.

[181] Buya AB, Beloqui A, Memvanga PB, Préat V (2020). Self-nano-emulsifying drug-delivery systems: from the development to the current applications and challenges in oral drug delivery. Pharmaceutics.

[182] Arts JHE, Hadi M, Keene AM (2014). A critical appraisal of existing concepts for the grouping of nanomaterials. Regul Toxicol Pharmacol.

[183] Mohammadi Z, Jafari SM (2021). Regulatory principles on food nanoparticles legislated by Asian and Oceanian countries. In: Safety and Regulatory Issues of Nanoencapsulated Food Ingredients. Cambridge, MA: Academic Press.

[184] Falkner R, Jaspers N (2012). Regulating nanotechnologies: risk, uncertainty and the global governance gap. Glob Environ Politics.

[185] US Food, Drug Administration (FDA) (2024). Considering whether an FDA-regulated product involves the application of nanotechnology. Silver Spring: FDA.

[186] Mühlebach S (2018). Regulatory challenges of nanomedicines and their follow-on versions: a generic or similar approach?. Adv Drug Deliv Rev.

[187] Ventola CL (2012). The nanomedicine revolution: part 3: regulatory and safety challenges. P T.

[188] Nardin I, Köllner S (2019). Successful development of oral SEDDS: screening of excipients from the industrial point of view. Adv Drug Deliv Rev.

